# Effect of evidence updates on key determinants of measles vaccination impact: a DynaMICE modelling study in ten high-burden countries

**DOI:** 10.1186/s12916-021-02157-4

**Published:** 2021-11-17

**Authors:** Han Fu, Kaja Abbas, Petra Klepac, Kevin van Zandvoort, Hira Tanvir, Allison Portnoy, Mark Jit

**Affiliations:** 1 Department of Infectious Disease Epidemiology, London School of Hygiene & Tropical Medicine, London, UK; 2 Public Health Foundation of India, New Delhi, India; 3 International Vaccine Institute, Seoul, South Korea; 4 Department of Applied Mathematics and Theoretical Physics, University of Cambridge, Cambridge, UK; 5 Center for Health Decision Science, Harvard T.H. Chan School of Public Health, Boston, MA USA; 6 Modelling and Economics Unit, Public Health England, London, UK; 7 School of Public Health, University of Hong Kong, Hong Kong, SAR China

**Keywords:** Measles, Vaccination impact, Transmission dynamic model, Routine vaccination, Supplementary immunisation activity

## Abstract

**Background:**

Model-based estimates of measles burden and the impact of measles-containing vaccine (MCV) are crucial for global health priority setting. Recently, evidence from systematic reviews and database analyses have improved our understanding of key determinants of MCV impact. We explore how representations of these determinants affect model-based estimation of vaccination impact in ten countries with the highest measles burden.

**Methods:**

Using Dynamic Measles Immunisation Calculation Engine (DynaMICE), we modelled the effect of evidence updates for five determinants of MCV impact: case-fatality risk, contact patterns, age-dependent vaccine efficacy, the delivery of supplementary immunisation activities (SIAs) to zero-dose children, and the basic reproduction number. We assessed the incremental vaccination impact of the first (MCV1) and second (MCV2) doses of routine immunisation and SIAs, using metrics of total vaccine-averted cases, deaths, and disability-adjusted life years (DALYs) over 2000–2050. We also conducted a scenario capturing the effect of COVID-19 related disruptions on measles burden and vaccination impact.

**Results:**

Incorporated with the updated data sources, DynaMICE projected 253 million measles cases, 3.8 million deaths and 233 million DALYs incurred over 2000–2050 in the ten high-burden countries when MCV1, MCV2, and SIA doses were implemented. Compared to no vaccination, MCV1 contributed to 66% reduction in cumulative measles cases, while MCV2 and SIAs reduced this further to 90%. Among the updated determinants, shifting from fixed to linearly-varying vaccine efficacy by age and from static to time-varying case-fatality risks had the biggest effect on MCV impact. While varying the basic reproduction number showed a limited effect, updates on the other four determinants together resulted in an overall reduction of vaccination impact by 0.58%, 26.2%, and 26.7% for cases, deaths, and DALYs averted, respectively. COVID-19 related disruptions to measles vaccination are not likely to change the influence of these determinants on MCV impact, but may lead to a 3% increase in cases over 2000–2050.

**Conclusions:**

Incorporating updated evidence particularly on vaccine efficacy and case-fatality risk reduces estimates of vaccination impact moderately, but its overall impact remains considerable. High MCV coverage through both routine immunisation and SIAs remains essential for achieving and maintaining low incidence in high measles burden settings.

**Supplementary Information:**

The online version contains supplementary material available at 10.1186/s12916-021-02157-4.

## Background

Measles is a highly contagious disease that may result in severe morbidity and mortality, particularly in young children and in settings with poor access to treatment. Vaccination is a safe and effective measure for measles prevention and control, as seen in high-income countries since its first licensure in 1961 [1]. The optimal age range of the first dose of measles-containing vaccine (MCV1) depends on the local variation in seasonality, birth rate, and access to care [2]. In settings with ongoing measles transmission, the World Health Organization (WHO) recommends delivering MCV1 to children of 9 months old, and following up with the second dose (MCV2) for children at 15–18 months old [3]. In addition, among countries with weak health systems, supplementary immunisation activities (SIAs) through vaccination campaigns are highly effective in protecting under-immunised and zero-dose children by closing immunity gaps and interrupting measles transmission [3]. Vaccination contributes to the establishment of high levels of population immunity required for measles elimination, which is verified by the absence of endemic measles transmission for at least 36 months [4]. In 2011, the Global Measles and Rubella Strategic Plan was set up with a goal to achieve measles elimination in at least five WHO regions by the end of 2020 [5]. However, coverage of MCV1 has stagnated since 2010 in many countries and has been set back in 2020 due to routine immunisation service disruptions and mass vaccination campaign suspensions caused by the COVID-19 pandemic [6]. This has increased immunity gaps and the risk of measles outbreaks.

Strategic investments to improve measles vaccine coverage globally are partially informed by model-based estimates of measles burden and the impact of vaccination. An analysis conducted by the Vaccine Impact Modelling Consortium found that 57% of all vaccine-related mortality reduction was due to measles vaccination in 98 low- and middle-income countries (LMICs) between 2000 and 2019 [7]. An updated analysis for 112 LMICs found that 47 million measles deaths were estimated to be averted from vaccination activities occurring between 2000 and 2030 [8]. However, such estimates are highly dependent on our knowledge of key determinants of measles incidence and mortality as well as vaccination impact. Over the past decade, there have been substantial advances to our knowledge of measles case-fatality risks (CFRs) [9], social contacts driving person-to-person infection transmission [10, 11], age-related vaccine efficacy [12], the ability of SIAs to reach zero-dose populations [13], and measles basic reproduction numbers [14]. Nonetheless, how this additional evidence on epidemiological, behavioural, and programmatic determinants affects estimates of vaccination impact has never been systematically explored.

In this study, we investigated the extent to which recent evidence updates about these determinants affects model-based estimates of measles burden and vaccination impact. To do this, we used the Dynamic Measles Immunization Calculation Engine (DynaMICE), a population-based dynamic model of measles transmission that has been used to inform vaccination impact [7]. We incorporated the updated data sources of these determinants into the DynaMICE model and assessed their individual and combined effects on the estimation of MCV impact and the development of effective vaccination strategies.

## Methods

### DynaMICE model

**Fig. 1:**
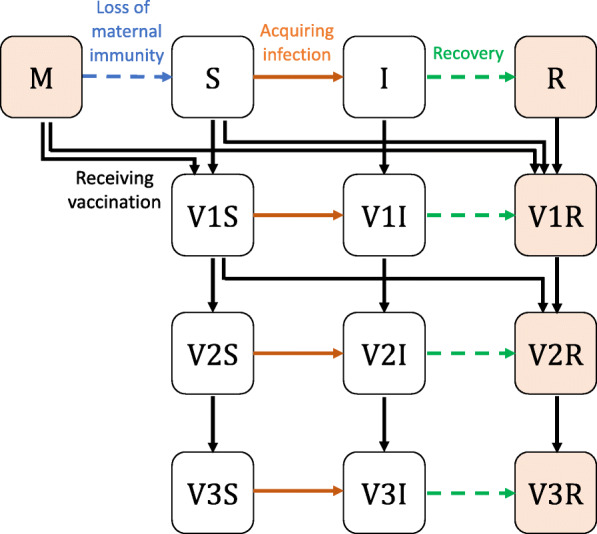
DynaMICE—Dynamic Measles Immunisation Calculation Engine. Model structure of DynaMICE for a single age stratum is presented. Individuals can be divided into 13 mutually exclusive states: M-maternally immune, S-susceptible, I-infectious, R-recovered. V1, V2, and V3 denote the received number of 1, 2, and 3 and more vaccine doses respectively. Individuals with natural or vaccine-acquired immunity (orange shaded squares) are not susceptible to measles. Arrows denote the change between two states over the transmission and progression of measles. In this model, births only add to the youngest age group. For clarity of presentation, ageing and death are not shown

DynaMICE is an age-structured compartmental transmission model designed to assess the impact of measles vaccination globally. Susceptible individuals become infected after effective contact with an infectious person and remain immune once they recover from their infection. Infants are born with or without maternal antibodies, depending on the immunity of their mothers. The population is further divided according to their received number of measles-containing vaccine doses (see Fig. 1). The age structure is composed of weekly age classes for the first 3 years of age, with annual age classes thereafter up to 100 years. The force of infection is calculated by multiplying an age-dependent per-capita contact rate with the total number of infectious people in the population and the probability of transmission per contact. The latter is calculated by scaling the next-generation matrix to reach a target basic reproduction number (R_0_) of 16, and assuming the average duration of infectiousness is 14 days. Annual seasonality of measles transmission was also incorporated into the model structure. Maternal immunity was assumed to last for an average of 6 months after birth [15], while individuals who recover from measles disease or acquire effective vaccine protection develop lifelong immunity. Measles deaths are calculated by applying an age-specific CFR to the incidence of cases. The model was coded in R and Fortran-95, and the code is available in https://github.com/lshtm-vimc/dynamice. Details of model equations and parameters are included in Additional file 1: Table S1 and Note S1, and also described in previously published studies [7, 16].

### Measles vaccination strategies

**Fig. 2:**
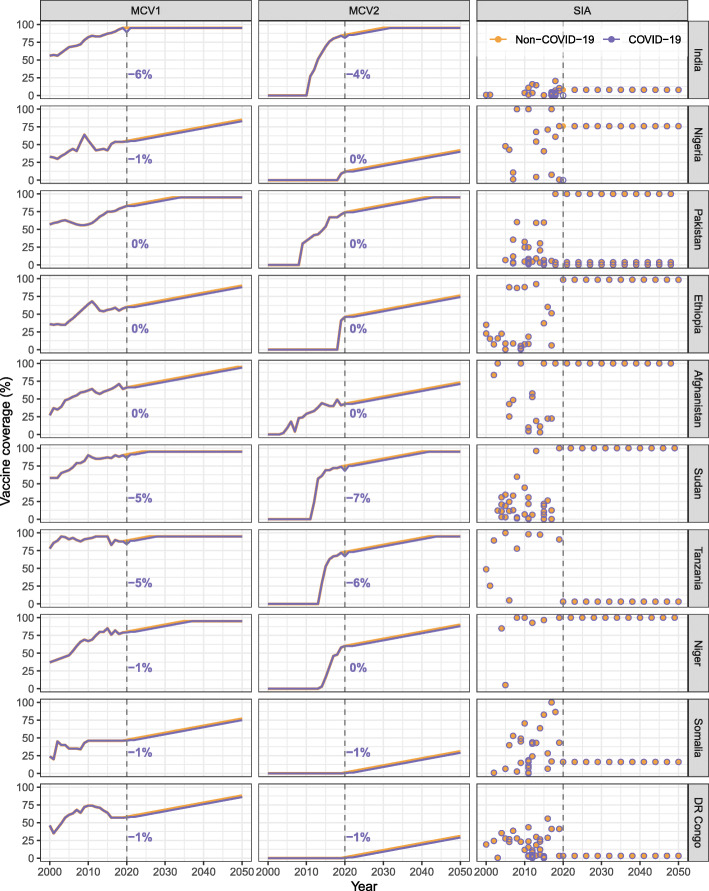
Measles vaccine coverage by dose and country, 2000–2050. Historical coverage data by dose and country were obtained from the WHO databases, while future trends were assumed to increase by 1% every year for the first (MCV1) and second (MCV2) doses delivered through routine programmes. Supplementary immunisation activities (SIAs) were assumed to occur every 3 years in the future projection, with the coverage seen in the most recent year. Multiple rounds of SIAs may happen within a single year in a country. Colours of lines and circles represent different assumptions on the effect of COVID-19 related disruptions. Vertical dashed lines mark the year 2000 and the numbers next to the lines show the absolute difference in MCV1 and MCV2 coverage estimates in the COVID-19 scenario compared to the baseline

In the DynaMICE model, vaccination can be delivered to any age or range of ages and in either routine vaccination (MCV1, MCV2) or SIAs. We assessed a range of model parameterisations from the perspective of four vaccination strategies: (1) no-vaccination, (2) MCV1 only, (3) MCV1 and MCV2, and (4) MCV1, MCV2, and SIAs. We assumed that the implementation of MCV1 and MCV2 complies fully with the WHO-recommended schedule [3], in which vaccine doses are delivered to children aged at 39 weeks (9 months) and 72 weeks (median of 15–18 months), respectively. MCV2 is delivered to children who have received one dose of measles vaccine. In addition to routine immunisation, SIAs target children of different age groups and occur at different intervals depending on settings. For each country, we adopted WHO-UNICEF estimates for historical coverage of routine immunisation [17] and calculated national SIA coverages by the number of doses delivered and the total number of population in a country (see Additional file 1: Note S2) [18]. Future vaccine coverage for routine immunisation was projected to increase by 1% per year, up to a maximum of 95% [7], while SIAs were assumed to take place every 3 years from the last recorded year with the same target population and coverage (see the following “COVID-19 related disruptions to measles vaccination” section and Fig. 2). We modelled vaccine efficacy as all-or-nothing, that is, offering complete protection to a defined proportion of the vaccinated population, and no protection to the remainder of the vaccinated population. The efficacy for the first dose was assumed to depend on the age of vaccination (with further explanation in the later sections), while the combined first and second dose was assumed to have 98% efficacy [19]. No additional protection was assumed to be received from any further doses of measles vaccine, and immunity was assumed to be lifelong.

### Measles vaccination impact metrics

We modelled measles transmission and vaccination impact in ten highest measles burden countries in 2000 – India, Nigeria, Pakistan, Ethiopia, Afghanistan, Sudan, Tanzania, Niger, Somalia, and Democratic Republic of the Congo (DR Congo), together accounting for more than 65% of global measles mortality in that year [20]. For each country and vaccination strategy, we projected time trends and age distributions of measles cases, deaths, and disability-adjusted life years (DALYs). We estimated the number of new infections, defined as incident measles cases, over 2000–2050, to estimate the historical impact of vaccination from 2000 to 2020, and to project the potential impact that continued coverage improvements may have after 2020. Measles-related deaths were calculated by multiplying the estimated number of measles cases with the specified age-specific CFR [9, 21]. DALYs for age group *a* at year *k* in country *c* were further calculated by:

$$ {\mathrm{DALY}}_{a,k,c}=\left({X}_{a,k,c}-{Y}_{a,k,c}\right)\times \omega +{Y}_{a,k,c}\times {L}_{a,k,c}, $$

where *X*_*a*, *k*, *c*_ and *Y*_*a*, *k*, *c*_ represent the number of cases and deaths, respectively; *ω* denotes the product of disability weight [22] and length of illness, equal to 0.002; and *L*_*a*, *k*, *c*_ denotes the remaining life expectancy at age of death [23]. No time discounting was applied to the DALY calculation. We also assessed the calendar year for a country to have conditions suitable to achieve measles elimination, based on an approximate criterion—measles incidence has been maintained at < 1 case per million population for at least three consecutive years [4, 24]. In assessing the key determinants of measles vaccination impact, we calculated cumulative vaccine-averted burden over 2000–2050 by comparing the vaccination strategies to the baseline with no vaccination.

### Evaluation of determinants

**Fig. 3:**
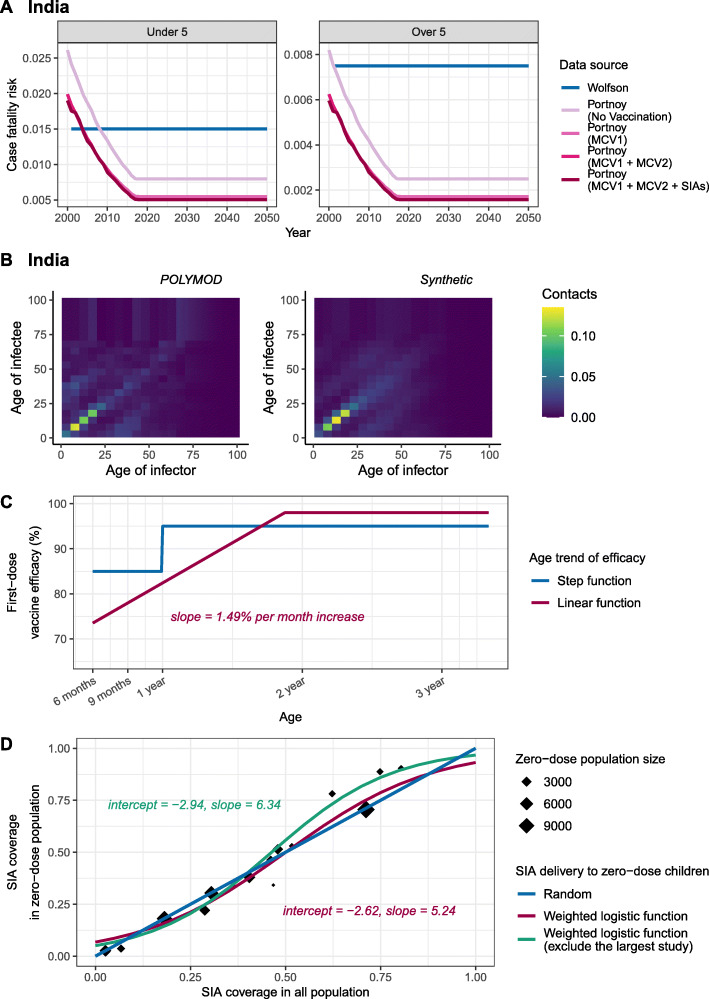
Updates for key determinants of measles vaccination. Historical and updated data sources for the four determinants are compared, including **A** case-fatality risk, **B** social contact patterns, **C** age-dependent vaccine efficacy, and **D** SIA coverage in zero-dose population. For country-specific assumptions like **A** and **B**, we demonstrated the data in India (the country with the highest measles mortality in 2000) and respectively include data in other countries in Additional file 1: Figure S1 and S2

We updated the data sources for five key determinants of vaccination impact in DynaMICE: case-fatality risk, social contact patterns, age-dependent vaccine efficacy, proportion of zero-dose children reached by SIAs, and basic reproduction numbers (Fig. 3). For the first four determinants, we first modelled a ‘base’ scenario based on previously adopted data sources [7, 16] and then altered each determinant individually to reflect recent evidence updates. We also evaluated a ‘full-update’ scenario that includes all the updated data sources for four of the determinants (Table 1) and examined the resulting changes in measles vaccination impact. For the fifth determinant, we explored a wide range of R_0_ values, based on a systematic review [14] which reveals a higher variability of measles transmissibility compared to what was previously known.

**Table 1:** Scenarios for evidence updates of key determinants of measles vaccination impact. Assumptions for four key determinants used in each scenario and their data sources are summarised. Abbreviations: *CFR* case-fatality risk, *SIA* supplementary immunisation activity

Scenarios	Data sources and assumptions for determinants
Base	With data sources used previously [7, 16]: • CFRs: country-specific, time-invariant estimates for under 5 years old from Wolfson [21] and halving such estimates for older than 5 years old. • Contact patterns: physical contact matrix from POLYMOD Great Britain [25]. • Age-dependent first-dose vaccine efficacy: step function which assumes 85% for under one year old and 95% for over 1 year old [19]. • SIA coverage in zero-dose population: equal coverage in population with or without previous vaccination (random distribution of SIA doses).
(A) CFR, Portnoy’s method	‘Base’ scenario, with updated CFRs: country-specific, time-varying, incidence-related estimates for under and over 5 years old from Portnoy et al. [9]
(B) Contact patterns, synthetic matrices	‘Base’ scenario, with update for contact patterns: country-specific synthetic matrices [10, 11]
(B’) Contact patterns, proportional mixing	‘Base’ scenario, with contact probabilities proportional to the age distribution of population
(B”) Contact pattern, uniform mixing	‘Base’ scenario, with uniform contact probabilities across age groups
(C) Age-dependent first-dose vaccine efficacy, linear trend	‘Base’ scenario, with updated first-dose vaccine efficacy: efficacy as a linear function of age with an increase of 1.49% per month of age, from 68% at birth to 98% at highest [12]
(D) SIA coverage in zero-dose population, dependency on previous vaccination	‘Base’ scenario, with update for SIA coverage in zero-dose population: informed by a weighted logistic function fitted to all available surveys [13]
(D’) SIA coverage in zero-dose population, dependency on previous vaccination, excluding the largest survey	‘Base’ scenario, with update for SIA coverage in zero-dose population: informed by a weighted logistic function fitted to available surveys except for the one with the largest sample size (Indonesia, 2002) [13]
(D”) SIA coverage in zero-dose population, 7.7% never reached	‘Base’ scenario, with random delivery of SIA doses except for an isolated 7.7% of the target population that are assumed to never receive any measles vaccine dose [26]

#### Case-fatality risk

The impact of measles vaccination using two sets of global CFR estimates was compared (Fig. 3A, and Additional file 1: Figure S1): (i) an earlier review of 58 publications by Wolfson et al. [21], which provided time-invariant CFRs for children under 5 years old in different regions, with a further assumption that CFR was halved for children over 5 years old [27]; and (ii) an updated review of 124 publications by Portnoy et al. [9], which estimated country-specific, time-varying CFRs for children under 5 and over 5 years old, using a log-linear model with covariates such as local measles attack rate, under-five mortality, calendar year, and percentage of population living in urban areas. For future projection, we assumed that CFRs remained stable at their 2018 levels over 2019–2050, in consideration of uncertain changes in future improvements of healthcare and vaccination programmes as well as the potential impact arising from the COVID-19 pandemic. The CFR estimates used in this study were also used in a modelling analysis of the impact of COVID-19 disruption to measles vaccination [6].

#### Social contact patterns

Our original study used the contact matrix adapted from the POLYMOD study [25] to represent age-dependent mixing patterns in all countries, because it showed sensible characterisation of measles transmission. Since then, several contact surveys in LMICs have been conducted; based on these updated surveys, country-specific demographics in 2020, and the most recent data for household structure, labour force, and school enrolment, synthetic contact matrices have been conducted for most countries [10, 11]. With these two types of social mixing matrices (Fig. 3B, and Additional file 1: Figure S2), we additionally considered two simple assumptions that are not based on empirical surveys: (i) the POLYMOD contact matrix from Great Britain, (ii) synthetic country-specific contact matrices, (iii) a matrix with contacts proportional to the age distribution of the population, and (iv) a uniform matrix with no age-dependency in mixing (see Additional file 1: Note S3). All the age-dependent matrices were normalised to reflect the assigned scale of R_0_, so the model results can be properly compared.

#### Age-dependent vaccine efficacy

As shown in Fig. 3C, we compared two assumptions for the age trend of first-dose vaccine efficacy: (i) a simplified step-change from 85% to 95% at a cut-off of 1 year old [19]; and (ii) a linear increase of 1.49% for every increase in month of age, derived from a recent systematic review of 33 measles vaccine efficacy studies in measles-endemic settings [12]. We capped the vaccine efficacy for the first dose at 98%, which is equivalent to the level of protection provided by two doses of MCV [19].

#### Proportion of zero-dose children reached by SIAs

Based on a previous exploration of the population reached by SIAs using Demographic and Health Surveys data for 14 countries [13], we fit a weighted logistic function to describe the association between the SIA coverages in total population and in zero-dose children (Fig. 3D). We then calculated the SIA doses given to the population with previous vaccination history (see Additional file 1: Note S1), by subtracting those doses received by zero-dose population from the total doses reported in the WHO data [18]. We compared this association to a random distribution of SIA doses in the ‘base’ scenario, where SIA doses are delivered to the target population regardless of their measles vaccination history. To assess the robustness of the weighted logistic function in representing the delivery of SIA, we refitted the function after excluding the survey with the largest sample size (also the oldest survey in the dataset), and then evaluated the vaccination impact. In addition, we assessed an alternative assumption that 7.7% of children are never reached by SIAs while the rest of the population are given SIAs randomly, using an approximation based on the prevalence of children who had not received any dose of MCV and other three essential childhood vaccines in a recent analysis [26].

#### Basic reproduction number

We assumed an R_0_ of 16 in the main analysis, according to a systematic review that included studies in LMICs in the vaccine era [14]. We further examined measles burden and vaccination impact with R_0_ values of 12, 20, and 24, to address the right-skewed distribution of estimates observed in the review [14].

### COVID-19 related disruptions to measles vaccination

In 2020, the WHO-UNICEF estimates report an absolute change in MCV1 and MCV2 coverages from − 6% to 5% (compared to 2019) across different countries [17], where the country variation is likely due to a mixed effect of COVID-19 service disruptions and ongoing efforts for immunisation programme expansion. To understand the potential effect of the COVID-19 disruptions on measles burden and vaccination impact, we examined two vaccine coverage scenarios (see Fig. 2):

(i) a ‘Non-COVID-19 scenario’ as the base case, considering there is still great uncertainty around how the impact of COVID-19 disruptions will last and how immunisation programmes will resume. Without COVID-19 disruptions, we assumed a 1% absolute coverage increase a year from 2019 and up to 95%, based on WHO-UNICEF routine coverage figures. For countries reporting a growth in the coverage estimates from 2019 to 2020, we took the 2020 estimates and assumed the annual 1% coverage increase to begin from 2020. SIAs are assumed to proceed as planned in 2020 for six countries (India, Nigeria, Ethiopia, Tanzania, Somalia, and DR Congo), with either their actual coverage in 2020 (if the SIA did indeed happen), or the coverage of the most recent SIA for similar target group and purpose in that country (if the SIA did not happen) [18].
(ii) a ‘COVID-19 scenario’ based on the 2020 WHO-UNICEF routine coverage estimates [17]. In 2021 and 2022, routine coverage in each country is assumed to return to its 2020 level in the ‘Non-COVID-19’ scenario [28]. The expansion for routine immunisation programme by 1% absolute coverage increase a year is delayed to 2023. For campaign vaccination, COVID-19 disruptions led to the cancellation of SIAs in India and Nigeria, two of the six countries planned for SIAs in 2020 [18], but future SIAs are assumed to be unaffected.

In addition, we assumed that measles CFRs are the same in both scenarios and are estimated using measles incidence and vaccine coverage in the ‘Non-COVID-19’ scenario. While the decline in vaccine coverage and excess measles incidence in the ‘COVID-19’ scenario are associated with increased measles CFR estimates, the relative increase was estimated to be less than 1%, according to the prediction model applied in Portnoy’s CFR estimates [9].

## Results

**Fig. 4:**
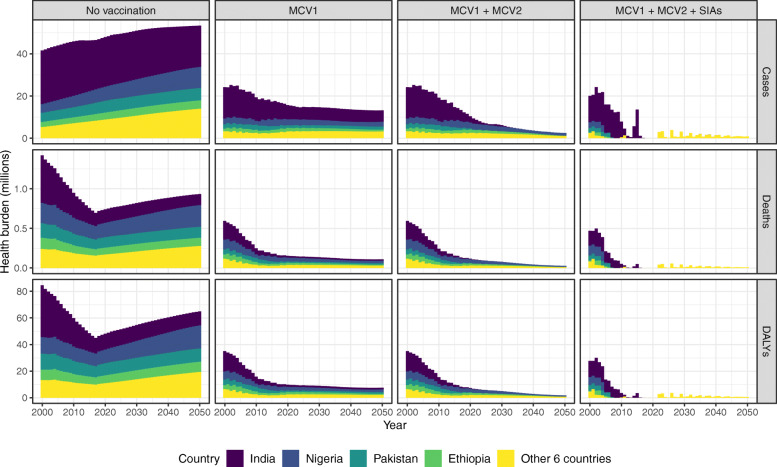
Measles vaccination impact by calendar year. Measles cases, deaths, and disability-adjusted life years by vaccination strategies and countries are presented based on the model results of the ‘full-update’ scenario that includes all the updates of key determinants. In India, Nigeria, Pakistan, Ethiopia, and other 6 countries (Afghanistan, Sudan, Tanzania, Niger, Somalia, DR Congo) with high measles burden, a substantial reduction of measles burden can be attributed to MCV1, while MCV2 and SIAs contribute to the maintenance of low-level measles transmission. Abbreviations: *MCV1* first routine dose of measles-containing vaccine, *MCV2* second routine dose of measles-containing vaccine, *SIAs* supplementary immunisation activities

Figure 4 shows model projections of annual measles incidence over 2000–2050 under different vaccination strategies in India, Nigeria, Pakistan, Ethiopia, and other six high-burden counties in the ‘full-update’ scenario that uses the most recent evidence for four determinants of vaccination impact and assumes no COVID-19 related disruptions. Under the implementation of MCV1, MCV2, and SIAs, 253 million measles cases, 3.8 million deaths, and 233 million DALYs are projected over the 51 years in the ten countries. In the COVID-19 scenario, an additional 7.1 million cases, 109 thousand deaths, and 6.7 million DALYs are projected over 2000–2050, corresponding to a nearly 3% increase in measles burden (Table 2). Among the top ten high-burden countries, India contributes the most to overall measles cases (47% with no vaccination) but a smaller proportion of overall deaths (26% with no vaccination), because of its relatively low CFR compared to other countries (see Additional file 1: Figure S1) [9].

**Table 2:** COVID-19-related disruptions to measles burden and vaccination impact. Total burden (cases, deaths, and DALYs) and vaccine impact (cases, deaths, and DALYs averted) in ten high measles burden countries over 2000–2050 are presented, based on coverage estimates without and with disruptions caused by COVID-19. Proportionate changes due to COVID-19 disruptions are shown in parentheses. Model scenarios in this analysis were conducted under the ‘full-update’ model, with the assumption of R_0_ = 16 and implementation of MCV1, MCV2, and SIA. Abbreviations: *DALY* disability-adjusted life years, *MCV1* first routine dose of measles-containing vaccine, *MCV2* second routine dose of measles-containing vaccine, *SIA* supplementary immunisation activity

	Measles burden over 2000–2050			Vaccine impact over 2000–2050	
Measurements (millions)	Non-COVID-19 scenario	COVID-19 scenario	Additional burden due to COVID-19	Non-COVID-19 scenario	COVID-19 scenario
Cases	253.43 (ref)	260.56 (+ 2.81%)	7.12	2243.81 (ref)	2236.69 (− 0.32%)
Deaths	3.76 (ref)	3.86 (+ 2.90%)	0.109	42.64 (ref)	42.53 (− 0.26%)
DALYs	233.43 (ref)	240.13 (+ 2.87%)	6.70	2797.73 (ref)	2791.03 (− 0.24%)

Model results show the important roles of routine immunisation and SIAs in measles control. Of these vaccination activities, the first dose provided through routine programmes (MCV1) contributes the most to burden reduction. Based on the ‘full-update’ model, cumulative measles cases over 2000–2050 are projected to reduce by 66% with MCV1 alone, 78% with a second routine dose (MCV1 and MCV2), and 90% with further inclusion of supplementary doses (MCV1, MCV2, and SIAs). The incremental impact of MCV2 and SIA doses is not as large as MCV1 but essential in maintaining a low level of measles transmission and preventing the periodical occurrence of large outbreaks as the number of susceptibles accumulates. Measles incidence is projected to be less than one case per million for at least three consecutive years by 2050 in Pakistan, Ethiopia, Sudan, and Niger, with high coverage of both MCV2 and SIA doses (see Additional file 2: Table S2). A low level of measles incidence could still be attained by either a high MCV2 coverage (India, Tanzania) or a high SIA coverage (Nigeria, Afghanistan), where the two vaccination activities could compensate for the performance of each other. Conversely, Somalia and DR Congo are not likely to maintain this low level of measles incidence prior to 2050, due to their limited coverages of MCV1, MCV2 and SIA doses.

**Fig. 5:**
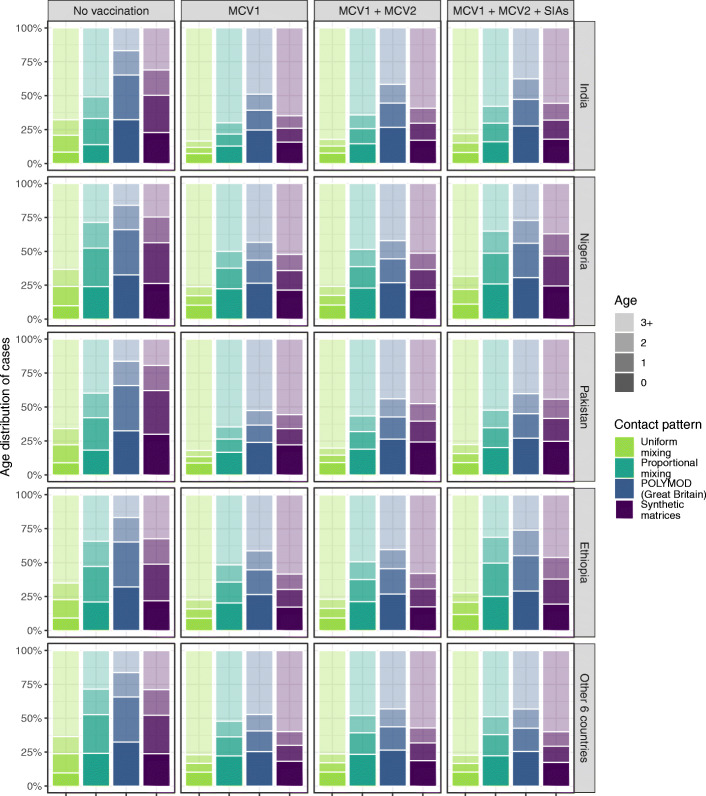
Age distribution of cumulative measles cases over 2000–2050 by different contact patterns. Proportions of measles cases aged 0, 1, 2, and 3+ years in India, Nigeria, Pakistan, Ethiopia, and other 6 countries (Afghanistan, Sudan, Tanzania, Niger, Somalia, DR Congo) with high measles burden are presented, by the assumptions of uniform mixing, proportional mixing, POLYMOD Great Britain contact matrix, and country-specific synthetic contact matrices. Compared to uniform mixing, contact patterns that consider age-related mixing tend to result in a lower average age at infection. Based on the POLYMOD matrix, the projected cases are more concentrated in younger age groups compared to the other contact patterns. Abbreviations: *MCV1* first routine dose of measles-containing vaccine, *MCV2* second routine dose of measles-containing vaccine, *SIAs* supplementary immunisation activities

Table 3 presents model estimates of vaccination impact in terms of total averted cases, deaths, and DALYs over 2000–2050 in the scenarios listed in Table 1 for assessing updated evidence sources. Using the updated CFR estimates from Portnoy’s review instead of Wolfson’s review is the most influential single change, resulting in 17.0–22.9% reduction in averted deaths and DALYs. Using contact patterns from synthetic matrices or the POLYMOD Great Britain matrix results in similar estimates of vaccine-averted cases and deaths, lying within 3.2% of each other. Under the assumptions of proportional and uniform mixing patterns, the total number of vaccine-averted cases is similar to the estimate based on the POLYMOD matrix. However, the assumptions of uniform mixing and proportional mixing result in a 22.9–26.0% and 7.3–9.6% reduction in the number of averted deaths, respectively, due to the increase in the mean age of measles infection and death (Fig. 5). Applying a linear trend in vaccine efficacy by age instead of a step function results in a 2.0–10.4% decline in averted cases, depending on the vaccination strategy. In comparison to random delivery of SIA doses, the weak dependency between SIA doses and previous vaccination status is projected to produce a small increase in averted cases (0.26–0.40%), using either of the logistic functions derived from previous surveys. Nevertheless, a 6.1% reduction in vaccine-averted cases is projected when assuming that 7.7% of children are never reached by SIAs, where there are fewer zero-dose children being benefited from SIA protection (see Additional file 2: Figure S3). Overall, the evidence updates about the CFR estimates and age trend in first-dose vaccine efficacy have the largest influence on MCV impact.

**Table 3:** Averted measles cases, deaths, and DALYs by vaccination strategies and evaluation scenarios. Total vaccine-averted burden in ten high measles burden countries over 2000–2050 is presented, with proportionate change from the ‘base’ scenario shown in parentheses. All model scenarios were conducted under the assumption of R_0_ = 16. Details of data sources for key determinants assumed for each scenario can be found in Table 1. Abbreviations: *CFR* case-fatality risk, *DALY* disability-adjusted life years, *MCV1* first routine dose of measles-containing vaccine, *MCV2* second routine dose of measles-containing vaccine, *SIA* supplementary immunisation activity

Scenarios	MCV1			MCV1 + MCV2			MCV1 + MCV2 + SIAs		
	Cases, millions	Deaths, millions	DALYs, millions	Cases, millions	Deaths, millions	DALYs, millions	Cases, millions	Deaths, millions	DALYs, millions
Base	1802 (ref)	47.3 (ref)	3155 (ref)	2023 (ref)	50.8 (ref)	3396 (ref)	2257 (ref)	57.8 (ref)	3819 (ref)
(A) CFR, Portnoy’s method	–	39.3 (− 17.0%)	2589 (− 17.9%)	–	40.9 (− 19.6%)	2698 (− 20.6%)	–	44.9 (− 22.3%)	2944 (− 22.9%)
(B) Contact pattern, synthetic matrices	1824 (+ 1.2%)	46.4 (− 1.8%)	3099 (− 1.8%)	2024 (+ 0.04%)	49.4 (− 2.9%)	3296 (− 2.9%)	2265 (+ 0.36%)	56.0 (− 3.2%)	3697 (− 3.2%)
(B’) Contact pattern, proportional mixing	1815 (+0.69%)	43.9 (− 7.3%)	2913 (− 7.7%)	1982 (− 2.0%)	46.4 (− 8.8%)	3076 (− 9.4%)	2215 (− 1.8%)	52.6 (− 8.9%)	3454 (− 9.6%)
(B”) Contact pattern, uniform mixing	1813 (0.61%)	36.5 (− 22.9%)	2413 (− 23.5%)	1964 (− 2.9%)	38.3 (− 24.6%)	2527 (− 25.6%)	2225 (− 1.4%)	43.5 (− 24.7%)	2826 (− 26.0%)
(C) Age-dependent vaccine efficacy, linear trend	1615 (− 10.4%)	42.8 (− 9.6%)	2853 (− 9.6%)	1939 (− 4.2%)	48.4 (− 4.8%)	3241 (− 4.6%)	2213 (− 1.9%)	56.6 (− 2.1%)	3743 (− 2.0%)
(D) SIA delivery to zero-dose population, dependency on previous vaccination	–	–	–	–	–	–	2266 (+ 0.40%)	57.94 (+ 0.26%)	3829 (+ 0.26%)
(D’) SIA delivery to zero-dose population, dependency on previous vaccination, excluding the largest survey	–	–	–	–	–	–	2263 (+ 0.25%)	57.89 (+ 0.18%)	3826 (+ 0.18%)
(D”) SIA delivery to zero-dose population, 7.7% never reached	–	–	–	–	–	–	2118 (− 6.1%)	53.8 (− 6.9%)	3576 (− 6.4%)
Full-update	1646 (− 8.7%)	35.9 (− 24.2%)	2367 (− 25.0%)	1949 (− 3.7%)	38.1 (− 25.2%)	2518 (− 25.9%)	2244 (− 0.58%)	42.6 (− 26.2%)	2798 (− 26.7%)

The combined effect of all these evidence updates (‘full-update’ scenario) is a decline in vaccine-averted burden, with a 0.58–8.7% reduction in cases averted, and a 24.2–26.7% reduction in deaths and DALYs averted (Table 3). However, similar measles burden trends are seen using the ‘base’ and ‘full-update’ models, both with a fast decline in measles cases following the country-specific rollout of routine immunisation and SIAs (see Additional file 2: Figure S4). Country schedule for reaching and maintaining a low level of measles transmission is also similar (see Additional file 2: Table S2). In addition, there are no significant differences in how the updated determinants affect MCV impact between the base case and COVID-19 scenarios, although the total number of vaccine-averted cases is projected to be smaller in the COVID-19 scenario (see Table 2, and Additional file 2: Table S4).

Next, we compare the age distribution of cumulative measles cases over 2000–2050 under different assumptions for contact patterns. Figure 5 presents the proportions of measles cases from 0 (first year of life), 1, 2, and 3 years and above of age in India, Nigeria, Pakistan, Ethiopia, and other six high-burden countries. Similar age distributions of measles cases are shown across countries. The update with country-specific synthetic matrices results in an older average age of measles cases, compared to the POLYMOD matrix. The age distribution of measles cases is least concentrated in younger age groups under the assumption of uniform mixing. Without the implementation of vaccination, the proportions of cases older than 3 years old in each country are 16–17% with the POLYMOD matrix, 20–32% with synthetic matrices, 22–51% with proportional mixing, and 62–68% with uniform mixing. The increase in the mean age under the assumption of uniform mixing accounts for the reduced vaccination impact against deaths, since older measles cases are at a lower risk of death (see Additional file 1: Figure S1), even though there are more cases prevented by vaccination (Table 3). We also observe a shift towards an older average age at infection in any of the measles vaccination strategies, compared to the model results assuming no vaccination.

**Fig. 6:**
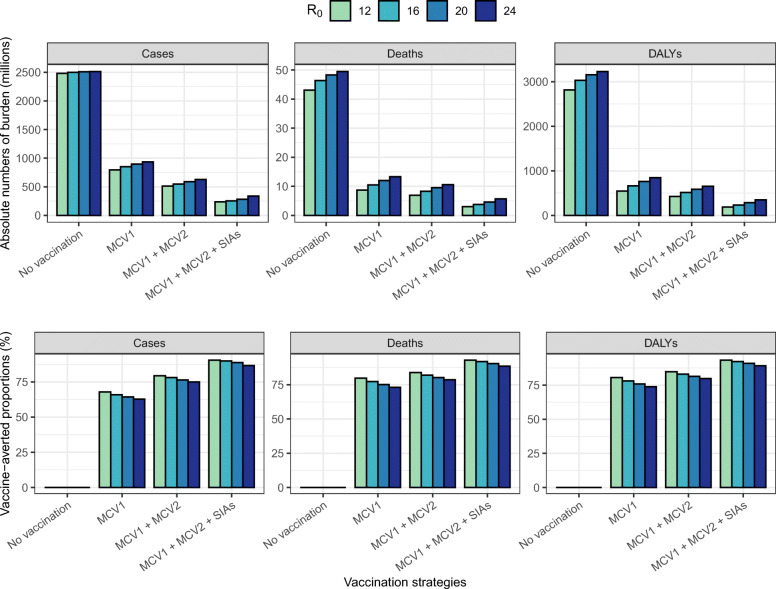
Cumulative measles burden over 2000–2050 by vaccination strategies and R_0_. The total number of cases, deaths, and DALYs (upper panel) and corresponding averted proportions (lower panel) in the four countries are compared by different values of R_0_. Despite the variability of R_0_, there is a limited effect on the vaccine impact estimates. Abbreviations: *DALY* disability-adjusted life years, *MCV1* first routine dose of measles-containing vaccine, *MCV2* second routine dose of measles-containing vaccine, *SIAs* supplementary immunisation activities

Figure 6 presents the absolute measles burden and vaccination impact over the evaluation period by different values of R_0_ in the ‘full-update’ scenario. A larger value of R_0_ led to a higher estimate of measles burden but a smaller vaccination impact. However, the overall impact of R_0_ is limited, with less than 5% of variation in vaccine-averted cases and deaths compared to the baseline R_0_. Increased burden driven by a larger R_0_ also delays the achievement of measles elimination, in particular for countries without high national coverage of SIAs (Additional file 2: Table S2). Nevertheless, our findings about the substantial contribution of measles vaccination and the relative impact of MCV1, MCV2 and SIA doses remain unchanged.

## Discussion

Using the DynaMICE model, we have considered the best available updated evidence and explored the influence of recent systematic reviews and database analyses on key determinants of measles vaccination impact: case-fatality risk, social contact patterns, age-dependent vaccine efficacy, proportion of zero-dose children reached by SIAs, and basic reproduction numbers. With the updated evidence, we project reduced estimates of vaccination impact marginally for cases averted and substantially for deaths and DALYs averted. As before, we find that the first vaccine dose delivered by routine immunisation programmes contributes to the largest reduction of measles burden, but the second routine dose and supplementary doses also provide important incremental benefits towards elimination. We find that updates to two of the determinants have the biggest changes in the vaccination impact: making the first-dose vaccine efficacy a linearly age-dependent function based on a recent review [12] decreases impact estimates by 2–10%, while using time-dependent and incidence-related CFRs [9] decreases them by 17–23%. Updated contact patterns, SIA dependency on previous vaccination history, and the basic reproduction number make relatively little difference to vaccination impact estimates. Overall, measles vaccination impact remains substantial despite these data updates, although the impact is relatively reduced when COVID-19-related disruptions are considered.

In the ten countries with the highest measles mortality in 2000, our model projections show varying trends of measles incidence under the vaccination strategy involving MCV1, MCV2 and SIAs doses. In Nigeria, Somalia, and DR Congo, measles transmission is projected to be suppressed for a few years followed by resurgence (see Additional file 2: Table S2). The difficulties faced by these countries result from having a lower coverage of routine immunisation (both MCV1 and MCV2) compared to the other countries, and their SIAs are not highly effective to close the measles immunity gaps (see Fig. 2). We did not model the possibility of measles elimination directly, since this would require consideration of case importation, contact tracing, and outbreak response, which are not captured by aggregate compartmental models like DynaMICE. However, the low measles incidence achieved by countries like India suggests that the measles elimination schedules aimed by the WHO regional office [29] may be possible if high coverage of both routine and campaign vaccination activities can be maintained in the long run (Additional file 2: Table S2, Figure S5 and S6).

Updating CFR estimates is the single most influential change among the determinants that we evaluated. The updated Portnoy’s CFRs start higher than Wolfson’s CFRs in 2000 but then decline (Additional file 1: Figure S1), whereas Wolfson’s CFRs are assumed to be time-invariant. Across the ten high-burden countries, a 17–23% reduction is seen in cumulative vaccine-averted deaths and DALYs over 2000–2050 with updated CFRs. Additionally, with MCV2 and SIA doses included in the vaccination strategy, the averted measles burden is projected to increase as a result of a relatively larger decline in Portnoy’s CFRs (Additional file 1: Figure S1). Referring to the log-linear model used for estimating CFRs in Portnoy’s method [9], we found measles attack rate, one of the covariates which can be largely reduced by vaccination, may explain the different levels of reduction in vaccination impact across vaccination strategies. Considering the importance of CFRs to estimates of measles burden and vaccination impact, understanding how CFRs may change in the future needs to be a key future research priority. In addition to case fatality, exploring the temporal variation in case severity and disability weight will improve the estimation of the measles disease burden.

The relationship between the first-dose vaccine efficacy and age at vaccination is another important determinant of MCV impact. According to the updated linear function [12], MCV1 given to children of 9 months old shows 78% efficacy, while 85% is assumed based on the step function used previously [19] (Fig. 3C). This update thus reduces our estimates of MCV1 impact; nevertheless, since the first-dose efficacy increases with age, SIAs that reach zero-dose population of an older age result in increased protection, which can partly offset the reduction in MCV1 protection (Table 3). This association between vaccine efficacy and age at vaccination will be useful for planning immunisation programmes, when combined with local epidemiology of measles transmission to maximise the MCV impact.

Like previous modelling studies [30, 31], our analysis shows the importance of age-dependent contact patterns in determining measles transmission and the impact of control strategies. Despite a small difference in the vaccination impact on preventing measles cases, a 23–26% reduction in deaths averted is projected under the assumption of uniform mixing, compared to the POLYMOD matrix (Table 1), due to the increase in the average age at measles infection (Fig. 5). Compared to uniform mixing, proportional mixing also relates to measles cases of a younger age, reflecting the age composition of local demographics that consists of a larger proportion of children in most high measles burden settings. On the other hand, there is little difference in model results between using the POLYMOD Great Britain matrix [25] and country-specific synthetic matrices [11]. Both matrices capture the main pattern of age-assortative mixing and thus led to similar estimates of vaccination impact. The slight difference in age distribution of measles cases could be attributed to the higher degree of age-assortativity in the POLYMOD Great Britain matrix (Additional file 1: Figure S2), which might be less representative of LMICs with more frequent contact between different age groups.

The proportion of zero-dose reached by SIAs is positively associated with the vaccine impact estimate because the efficacy of the first MCV dose is higher than the incremental protection of the second or further doses for already-vaccinated children. However, when countries reach a high MCV1 coverage and contain a small size of zero-dose children, the estimation of vaccine impact is less sensitive to the assumption of SIA dependency on vaccination status. This may partly explain the small MCV impact seen in the update on the proportion of zero-dose reached by SIAs (Table 3). There is also remaining uncertainty around the delivery of SIA doses. The fitted logistic function adopted in the update analysis indicates that zero-dose children are more likely to be reached when the coverage in the total population is between 50 and 85% (Fig. 3D), although the dependency is weak and the delivery of SIA doses is nearly random. However, this evidence update is based on surveys conducted during 2002–2008 [13], and more recent SIAs may have been better able to address subnational inequalities in vaccination coverage, e.g., by targeting rural areas or hard-to-reach populations [32]. There is also a lack of representativeness of geographical diversity, since those survey data were collected in 14 countries and might not include marginalised populations who are likely to have limited access to measles vaccination [13]. Thus, it is possible that a concerning fraction of children have been missed by both routine immunisation programmes and SIAs [26]. We evaluated this possibility in an alternative scenario with 7.7% of children never reached by SIAs and found a significant reduction in vaccination impact compared to random delivery (see Additional file 2: Figure S4). The ability of reaching zero-dose populations through SIAs has crucial implications for estimating MCV impact and planning immunisation activities, and therefore requires further research to clarify.

Our results present a substantial decline in measles cases and deaths over 2000–2019 in ten high-burden countries. The country rank of measles mortality in 2019 has changed and India is no longer the country with the highest burden (Additional file 2: Table S2). The same overall trends (declining burden over time) are seen in model-based estimates of measles incidence over the same period from Global Burden of Diseases 2019 by the Institute for Health Metrics and Evaluation (IHME) [20], although our results show lower absolute incidence estimates over time (see Additional file 2: Figure S5). The difference in the incidence estimates may stem from different approaches used in the two models. DynaMICE is a mechanistic SIR transmission model which translates vaccine coverage into measles incidence using age-dependent contact data. In contrast, IHME uses a statistical model which estimates measles incidence with covariates including MCV1, MCV2 and SIA coverage estimates, based on an assumption of additive effects [33]. Consequently, DynaMICE may underestimate measles incidence at high levels of routine and SIA coverage, when the majority of targeted age groups may be vaccinated with at least one dose. Furthermore, we compared the model outputs to the WHO case notifications provided by country health departments [34]. In Pakistan, Sudan, and Niger, our model estimates suggested that country-specific measles incidence is maintained below one per million prior to 2020 (Additional file 2: Table S2), while there were more than 2,000 cases notified in 2019. The inconsistency between model estimates and notification data at very low levels of incidence may be associated with outbreaks related to heterogeneous MCV coverage at the subnational level [32, 35], which are not precisely captured by a model based on national averages of vaccine coverage. Additionally, measles notification data need to be interpreted carefully, as these data may be affected by underreporting and time-dependent biases due to varying surveillance and diagnostic capacity [27].

Our analysis has some limitations. First, we acknowledge that vaccine coverage forecasts in both ‘Non-COVID-19’ and ‘COVID-19’ scenarios are speculative, because there is no way to predict future events that may lead to disruptions to MCV coverage (such as civil unrest or vaccine confidence crises). Second, we assumed that MCV1 is delivered promptly to children at 9 months old, which assumes perfect timeliness of the routine immunisation programme schedule. This assumption of perfect timeliness may not be realistic in most settings, where many children receive MCV1 earlier, or more likely, later than the targeted age [36]. Thus, setting-specific data on the precise age at vaccination could provide further insight into vaccination impact. Third, we assumed the same R_0_ across countries due to a lack of systematic data for estimating the country-specific transmissibility. Combining with the age-dependent contact patterns, measles models fitted to data on incidence and serology may be helpful to estimate the force of measles infection in each country [31]. Nonetheless, our model findings remain robust and useful, as we found in sensitivity analyses that the variations in R_0_ within the range 12–24 do not substantially affect the vaccination impact (Fig. 6). Fourth, we did not consider importation of measles cases from migration, which can be a major source of local outbreaks when domestic measles transmission has been largely eliminated [37]. Additionally, we assumed that vaccine coverage was uniform over the entire country. However, the countries we modelled all have subnational regions with substantially lower coverage than average, and which therefore sustain measles transmission even when it is eliminated in the rest of the country. Finally, while we recommend incorporating these evidence updates into measles models in the future analysis, uncertainties in the quality and model representation of the collected evidence should not be overlooked. For example, in our analysis, contact pattern was adjusted only for varying demographics over 2000–2050 (see Additional file 1: Note S2), while temporal changes in societal and behavioural factors were not considered because of the lack of longitudinal surveys and data. As more data and evidence on the key determinants of vaccination impacts are available, an updated review and data synthesis will be required.

## Conclusions

Recent advances in data and research have provided a better understanding of measles transmission and vaccination impact. Using the DynaMICE model, we have systematically assessed updates on the key determinants of measles epidemiology and vaccination in ten countries with historical high measles mortality. We identified that measles vaccination impact is sensitive to the assumptions on CFR and age-dependent vaccine efficacy and reassure the importance of age-dependent social contact structure. However, as we have demonstrated in this study, these evidence updates would not undermine the substantial contribution of measles vaccination. High coverage of both measles vaccine doses, either through routine or SIA vaccine delivery, is essential for meeting and maintaining the goals for measles elimination.

## Supplementary Information

**Additional file 1: Supplementary materials and methods.** Table S1–List of parameters in DynaMICE. Note S1–Model equations. Note S2–Calculating national SIA coverage. Note S3–Constructing contact patterns. Figure S1–Case-fatality risks using Wolfson’s and Portnoy’s approaches, 2000–2050. Figure S2–Standardised social contact matrices in ten high-burden countries.

**Additional file 2: Supplementary results.** Table S2–Calendar year at which low measles incidence is achieved. Table S3–National and total measles burden by selected scenarios and vaccine strategies. Table S4–Averted measles burden in consideration of COVID-19 related disruptions. Figure S3–Model estimates under different assumptions for SIA delivery to zero-dose children. Figure S4–Model estimates of measles cases in the ‘base’ and ‘full-update’ scenarios. Figure S5–Model estimates of (A) measles cases and (B) deaths over 2000–2050 under different assumptions of R_0_. Figure S6–Model estimates of (A) measles cases and (B) deaths over 2000–2050 under different assumptions of R_0_, assuming no SIAs after 2019.

## Data Availability

Publicly available datasets were used for this modelling analysis, including country-specific coverage data for measles vaccination from the World Health Organization (https://immunizationdata.who.int/pages/coverage/mcv.html; https://www.who.int/entity/immunization/monitoring_surveillance/data/Summary_Measles_SIAs.xls) and population statistics from the United Nations World Prospect Project 2019 (https://population.un.org/wpp/). Computer codes for simulating measles burden and vaccination strategies based on the DynaMICE model are available in https://github.com/lshtm-vimc/dynamice.

## Abbreviations

CFR: Case-fatality risk
DALY: Disability-adjusted life year
DynaMICE: Dynamic Measles Immunisation Calculation Engine
IHME: Institute for Health Metrics and Evaluation
LMICs: Low- and middle-income countries
MCV: Measles-containing vaccine
SIA: Supplementary immunisation activity
WHO: World Health Organization
