# Supplementary material for: Effect of evidence updates on key determinants of measles vaccination impact: a DynaMICE modelling study in ten high-burden countries
Source: BMC Med. 2021 Nov 17;19:281. doi: 10.1186/s12916-021-02157-4 (PMC8594955; doi:10.1186/s12916-021-02157-4)
Supplement: Supplementary file 1 — Additional file 1: Supplementary materials and methods. Table S1–List of parameters in DynaMICE. Note S1–Model equations. Note S2–Calculating national SIA coverage. Note S3–Constructing contact patterns. Figure S1–Case-fatality risks using Wolfson’s and Portnoy’s approaches, 2000–2050. Figure S2–Standardised social contact matrices in ten high-burden countries. [file 12916_2021_2157_MOESM1_ESM.pdf]

Additional file 1

Supplementary materials and methods for the manuscript entitled "Effect of evidence updates on key determinants of measles vaccination impact: a DynaMICE modelling study in ten high-burden countries"

Han Fu[1], Kaja Abbas[1,2,3], Petra Klepac[1,4], Kevin van Zandvoort[1], Hira Tanvir[1], Allison Portnoy[5], Mark Jit[1,6,7]

[1]Department of Infectious Disease Epidemiology, London School of Hygiene & Tropical Medicine, London, UK

[2]Public Health Foundation of India, New Delhi, India

[3]International Vaccine Institute, Seoul, South Korea

[4]Department of Applied Mathematics and Theoretical Physics, University of Cambridge, Cambridge, UK

[5]Center for Health Decision Science, Harvard T.H. Chan School of Public Health, Boston, MA, USA

[6]Modelling and Economics Unit, Public Health England, London, UK

[7]School of Public Health, University of Hong Kong, Hong Kong, SAR, China

Contents

Table S1. List of parameters in DynaMICE 2

Note S1. Model equations 3

Note S2. Calculating national SIA coverage 6

Note S3. Constructing contact patterns 6

Figure S1. Case-fatality risks using Wolfson's and Portnoy's approaches, 2000–2050. 8

Figure S2. Standardised social contact matrices in ten high-burden countries. 9

References 10

Table S1. List of parameters in DynaMICE

Symbol	Definition	Value	References
$\boldsymbol{C}_{a,a'}$	Standardised age-dependent contact matrix between a susceptible of age group a and an infectious case of age group a'	country-specific	Mossong et al. [1]<br>Prem et al. [2]
R_0	Basic reproduction number	16	Guerra et al. [3]<br>Varied in the sensitivity analysis
g	Recovering rate	1/14 d^{-1}	Strebel et al. [4]
m	Maternal immunity waning rate	1/0.5 y^{-1}	Lasting for an average of 6 months.
σ_a	Ageing rate of age group a, weekly for for 0, 1, and 2 years old and yearly for 3 to 100 years old	1/7 d^{-1} (weekly)<br>1 y^{-1} (annual)	Adjusted to match the age structure and timestep
ε	Amplification factor for seasonality	0.05	Assumed
ϕ	Two-dose vaccine efficacy	0.98	Sudfeld, Navar, and Halsey [5]
$\delta_{1,a}$	First-dose vaccine efficacy of age group a	0.64598+0.01485* age in months	Hughes et al. [6]<br>Determined by a linear function and assumed $\leq \phi$
$\delta_{2,a,t}$	Second-dose vaccine efficacy of age group a at time t	See Note S1: Part 2 of model equations	Depending on the proportion of effective protection among those who have first-dose vaccination
δ_3	Third-dose vaccine efficacy	0	Assumed
$v_{1,a,t}$	First-dose routine vaccination coverage of age group a	country-specific	WHO [7]
$v_{2,a,t}$	Second-dose routine vaccination coverage of age group a	country-specific	WHO [7]<br>Only delivered to 72-week-olds
$v_{3,a,t}$	SIA coverage in overall population of age group a at time t	country-specific	WHO [8]<br>See 'Note S2. Calculating national SIA coverage' section

Note S1. Model equations

We present the difference equations used in the DynaMICE model, first with the part that depicts measles transmission dynamics and followed by ageing and vaccination. Parameters in these equations are summarised in Table S1.

In the DynaMICE model, individuals in each age group are divided into 13 compartments to capture different states of measles dynamics. The total population of all the states in an age group a at time t is denoted as $N_{a,t}$. There are 254 age groups included in the model, with the first 156 groups representing the weekly age for those from 0 to 2 years old and the rest for the yearly age between 3 and 100 years old. The basic compartments for measles transmission include maternally immune ($M_{a,t}$), susceptible ($S_{a,t}$), infectious ($I_{a,t}$), and recovered ($R_{a,t}$) states; additional compartments are included to distinguish the first ($VS_{a,t}$, $VI_{a,t}$, $VR_{a,t}$), second ($V2S_{a,t}$, $V2I_{a,t}$, $V2R_{a,t}$), and third times ($V3S_{a,t}$, $V3I_{a,t}$, $V3R_{a,t}$) of an individual being targeted and reached by vaccination programmes.

$$N_{a,t} = M_{a,t} + S_{a,t} + I_{a,t} + R_{a,t} + VS_{a,t} + VI_{a,t} + VR_{a,t} + V2S_{a,t} + V2I_{a,t} + V2R_{a,t} + V3S_{a,t} + V3I_{a,t} + V3R_{a,t}$$

Part 1: Transmission dynamics

Measles transmission is governed by force of measles infection ($\lambda_{a,t}$), which consists of seasonal variation (ω_t), disease transmissibility (R_0), and standardised, age-dependent contact matrix ($\boldsymbol{C}_{a,a'}$).

$$\lambda_{a,t} = \omega_t R_0 g \sum_{a'=1}^{a'=254} \boldsymbol{C}_{a,a'} \frac{(I_{a',t} + VI_{a',t} + V2I_{a',t} + V3I_{a',t})}{N_{a',t}}$$

$$\omega_t = 1 + \varepsilon \sin(2\pi t)$$

$$S_{a,t+1} = S_{a,t} - \lambda_{a,t} S_{a,t}$$

$$I_{a,t+1} = I_{a,t} + \lambda_{a,t} S_{a,t} - g I_{a,t}$$

$$R_{a,t+1} = R_{a,t} + g I_{a,t}$$

$$VS_{a,t+1} = VS_{a,t} - \lambda_{a,t} VS_{a,t}$$

$$VI_{a,t+1} = VI_{a,t} + \lambda_{a,t} VS_{a,t} - g VI_{a,t}$$

$$VR_{a,t+1} = VR_{a,t} + g VI_{a,t}$$

$$V2S_{a,t+1} = V2S_{a,t} - \lambda_{a,t} V2S_{a,t}$$

$$V2I_{a,t+1} = V2I_{a,t} + \lambda_{a,t} V2S_{a,t} - g V2I_{a,t}$$

$$V2R_{a,t+1} = V2R_{a,t} + g V2I_{a,t}$$

$$V3S_{a,t+1} = V3S_{a,t} - \lambda_{a,t} V3S_{a,t}$$

$$V3I_{a,t+1} = V3I_{a,t} + \lambda_{a,t} V3S_{a,t} - g V3I_{a,t}$$

$$V3R_{a,t+1} = V3R_{a,t} + g V3I_{a,t}$$

Part 2: Ageing and vaccination implementation

Routine vaccination is delivered to the target age group at the same time as ageing occurs, while SIAs are delivered at the beginning of the selected years. SIAs conducted at subnational level are merged to generate a national coverage for model input. As Figure 3D in the main text shown, we applied a logistic function based on the SIA coverage in the overall target population ($v_{3,a,t}$) to address the dependency of SIAs on previous vaccination history by SIAs. We then compared the number of doses delivered to the zero-dose

population ($D_{zero,a,t}$) and total target population ($D_{all,a,t}$), and estimated the respective SIA coverages in populations with ($\kappa_{2,a,t}$) and without ($\kappa_{1,a,t}$) previous measles vaccination history.

$$D_{zero,a,t} = \left(\frac{e^{-2.6217+5.2383\, v_{3,a,t}}}{1+e^{-2.6217+5.2383\, v_{3,a,t}}}\right)\left(S_{a,t}+I_{a,t}+R_{a,t}\right)$$

$$D_{all,a,t} = v_{3,a}N_{a,t}$$

$$\kappa_{1,a,t} = \begin{cases} \dfrac{e^{-2.6217+5.2383\, v_{3,a,t}}}{1+e^{-2.6217+5.2383\, v_{3,a,t}}}, & D_{all,a,t} \geq D_{zero,a,t} \\ \dfrac{D_{all,a,t}}{D_{zero,a,t}} & , \; otherwise \end{cases}$$

$$\kappa_{2,a,t} = \begin{cases} \dfrac{D_{zero,a,t} - D_{all,a,t}}{N_{a,t} - \left(S_{a,t}+I_{a,t}+R_{a,t}\right)}, & D_{all,a,t} \geq D_{zero,a,t} \\ 0 & , \; otherwise \end{cases}$$

A compartment ($VEFF_{a,t+1}$), although not contributing to measles dynamics, is used to count the population size with effective first-dose vaccine protection, which is later used to determine the second-dose efficacy ($\delta_{2,a,t}$).

$$\begin{aligned} VEFF_{a,t+1} = {} & VEFF_{a,t} - \sigma_a VEFF_{a,t} + \sigma_a\left(1 - v_{2,a-1,t+1}\right)VEFF_{a-1,t} \\ & + \sigma_a \delta_{1,a}\left(v_{1,a-1,t+1} + v_{2,a-1,t+1} - v_{2,a-1,t+1}v_{1,a-1,t+1}\right)v_{1,a-1,t+1}S_{a-1,t+1} \end{aligned}$$

$$\rho_{a,t} = \frac{VEFF_{a,t}}{VS_{a,t}+VI_{a,t}+VR_{a,t}}$$

$$\delta_{2,a,t} = \begin{cases} \phi & , \; VS_{a,t}+VI_{a,t}+VR_{a,t} = 0 \\ \dfrac{\phi - \rho_{a,t}}{1-\rho_{a,t}}, & (VS_{a,t}+VI_{a,t}+VR_{a,t} \neq 0) \quad \wedge \quad (\phi > \rho_{a,t}) \\ 0 & , \; otherwise \end{cases}$$

$$M_{a,t+1} = M_{a,t} - \sigma_a M_{a,t} + \sigma_a\left(1 - v_{1,a-1,t+1}\right)M_{a-1,t} - mM_{a,t}$$

$$S_{a,t+1} = S_{a,t+1} - \sigma_a S_{a,t+1} + \sigma_a\left(1 - v_{1,a-1,t+1}\right)S_{a-1,t+1} + mM_{a,t} - \kappa_{1,a,t}S_{a,t+1}$$

$$I_{a,t+1} = I_{a,t+1} - \sigma_a I_{a,t+1} + \sigma_a\left(1 - v_{1,a-1,t+1}\right)I_{a-1,t+1} - \kappa_{1,a,t}I_{a,t+1}$$

$$R_{a,t+1} = R_{a,t+1} - \sigma_a R_{a,t+1} + \sigma_a\left(1 - v_{1,a-1,t+1}\right)R_{a,t+1} - \kappa_{1,a,t}R_{a,t+1}$$

$$\begin{aligned} VS_{a,t+1} = {} & VS_{a,t+1} - \sigma_a VS_{a,t+1} + \sigma_a\left(1 - v_{2,a-1,t+1}\right)VS_{a-1,t+1} + \sigma_a\left(1-\delta_{1,a}\right)v_{1,a-1,t+1}\left(S_{a-1,t+1} + M_{a-1,t}\right) \\ & + \kappa_{1,a,t}\left(1-\delta_{1,a}\right)S_{a,t+1} - \kappa_{2,a,t}VS_{a,t+1} \end{aligned}$$

$$\begin{aligned} VI_{a,t+1} = {} & VI_{a,t+1} - \sigma_a VI_{a,t+1} + \sigma_a\left(1 - v_{2,a-1,t+1}\right)VI_{a-1,t+1} + \sigma_a v_{1,a-1,t+1}I_{a-1,t+1} + \kappa_{1,a,t}I_{a,t+1} \\ & - \kappa_{2,a,t}VI_{a,t+1} \end{aligned}$$

$$\begin{aligned} VR_{a,t+1} = {} & VR_{a,t+1} - \sigma_a VR_{a,t+1} + \sigma_a\left(1 - v_{2,a-1,t+1}\right)VR_{a-1,t+1} + \sigma_a \delta_{1,a} v_{1,a-1,t+1}\left(S_{a-1,t+1} + M_{a-1,t}\right) \\ & + \sigma_a v_{1,a-1,t+1}R_{a-1,t+1} + \kappa_{1,a,t}R_{a,t+1} + \kappa_{1,a,t}\delta_{1,a}S_{a,t+1} - \kappa_{2,a,t}VR_{a,t+1} \end{aligned}$$

$$V2S_{a,t+1} = V2S_{a,t+1} - \sigma_a V2S_{a,t+1} + \sigma_a\big(1 - v_{2,a-1,t+1}\big)V2S_{a-1,t+1} + \sigma_a v_{2,a-1,t+1}\big(1 - \delta_{2,a,t+1}\big)VS_{a-1,t+1} + \kappa_{2,a,t}\big(1 - \delta_{2,a,t+1}\big)VS_{a,t+1} - \kappa_{2,a,t}V2S_{a,t+1}$$

$$V2I_{a,t+1} = V2I_{a,t+1} - \sigma_a V2I_{a,t+1} + \sigma_a\big(1 - v_{2,a-1,t+1}\big)VI_{a-1,t+1} + \kappa_{2,a,t}VI_{a,t+1} - \kappa_{2,a,t}V2I_{a,t+1}$$

$$V2R_{a,t+1} = V2R_{a,t+1} - \sigma_a V2R_{a,t+1} + \sigma_a\big(1 - v_{2,a-1,t+1}\big)V2R_{a-1,t+1} + \sigma_a v_{2,a-1,t+1}\delta_{2,a,t+1}VS_{a-1,t+1} + \sigma_a v_{2,a-1,t+1}VR_{a-1,t+1} + \kappa_{2,a,t}VR_{a,t+1} - \kappa_{2,a,t}V2R_{a,t+1}$$

$$V3S_{a,t+1} = V3S_{a,t+1} - \sigma_a V3S_{a,t+1} + \sigma_a V3S_{a-1,t+1} + \sigma_a v_{2,a-1,t+1}(1 - \delta_3)V2S_{a-1,t+1} + \kappa_{2,a,t}(1 - \delta_3)V2S_{a,t+1}$$

$$V3I_{a,t+1} = V3I_{a,t+1} - \sigma_a V3I_{a,t+1} + \sigma_a v_{2,a-1,t+1}V3I_{a-1,t+1} + \kappa_{2,a,t}V2I_{a,t+1}$$

$$V3R_{a,t+1} = V3R_{a,t+1} - \sigma_a V3R_{a,t+1} + \sigma_a V3R_{a-1,t+1} + \sigma_a v_{2,a-1,t+1}\delta_3 V2S_{a-1,t+1} + \sigma_a v_{2,a-1,t+1}V2R_{a-1,t+1} + \kappa_{2,a,t}V2R_{a,t+1} + \kappa_{2,a,t}\delta_3 V2S_{a,t+1}$$

For the youngest age group ($a = 1$), the above equations are specially modified and shown below. We assume newborns are either susceptible or maternally immune, using a proportion of immune population in child-bearing ages from 20 ($a0 = 174$) to 35 ($a1 = 189$) years old. No measles vaccination is scheduled to deliver to newborns.

$$M_{1,t+1} = M_{1,t} - \sigma_a M_{1,t} + \sigma_a \frac{\sum_{a=a0}^{a1} R_{a,t+1} + VR_{a,t+1} + V2R_{a,t+1} + V3R_{a,t+1}}{\sum_{a=a0}^{a1} S_{a,t+1} + VS_{a,t+1} + V2S_{a,t+1} + V3S_{a,t+1} + R_{a,t+1} + VR_{a,t+1} + V2R_{a,t+1} + V3R_{a,t+1}}$$

$$S_{1,t+1} = S_{1,t+1} - \sigma_a S_{1,t+1} + \sigma_a \frac{\sum_{a=a0}^{a1} S_{a,t+1} + VS_{a,t+1} + V2S_{a,t+1} + V3S_{a,t+1}}{\sum_{a=a0}^{a1} S_{a,t+1} + VS_{a,t+1} + V2S_{a,t+1} + V3S_{a,t+1} + R_{a,t+1} + VR_{a,t+1} + V2R_{a,t+1} + V3R_{a,t+1}}$$

$$VEFF_{1,t+1} = VEFF_{1,t+1} - \sigma_a VEFF_{1,t+1}$$

$$R_{1,t+1} = R_{1,t+1} - \sigma_a R_{1,t+1}$$

$$VS_{1,t+1} = VS_{1,t+1} - \sigma_a VS_{1,t+1}$$

$$VI_{1,t+1} = VI_{1,t+1} - \sigma_a VI_{1,t+1}$$

$$VR_{1,t+1} = VR_{1,t+1} - \sigma_a VR_{1,t+1}$$

$$V2S_{1,t+1} = V2S_{1,t+1} - \sigma_a V2S_{1,t+1}$$

$$V2I_{1,t+1} = V2I_{1,t+1} - \sigma_a V2I_{1,t+1}$$

$$V2R_{1,t+1} = V2R_{1,t+1} - \sigma_a V2R_{1,t+1}$$

$$V3S_{1,t+1} = V3S_{1,t+1} - \sigma_a V3S_{1,t+1}$$

$$V3I_{1,t+1} = V3I_{1,t+1} - \sigma_a V3I_{1,t+1}$$

$$V3R_{1,t+1} = V3R_{1,t+1} - \sigma_a V3R_{1,t+1}$$

Note S2. Calculating national SIA coverage

In the DynaMICE model, SIAs are implemented at the beginning month of each calendar year and at national level. To obtain the input for national coverages over time, we first extracted the number of population reached by historical SIAs and targeted ranges for the age groups from the WHO database [8]. We assumed that infants younger than 6 months old are not eligible to receive an SIA dose, if the lower bound of the targeted age group is not available in the WHO data. For SIAs engaged with 'school-age' population, we assumed children aged between 6 and 17 years old are targeted. In countries where there are multiple records of SIA events in the same calendar year, we assumed they are implemented with a one-month interval. The national number of population reached by SIAs was then divided by the national population size [9] for each age group, to calculate the time- and age-specific SIA coverages, denoted as $v_{3,a,t}$ in Table S1 and model equations. The coverages are capped to 100% and presented for the top ten countries with high measles mortality (Figure 2 in the main text).

Note S3. Constructing contact patterns

As shown in Part 1 of model equations in Note S1, we incorporated a contact matrix ($\boldsymbol{C}_{a,a'}$) in the DynaMICE model to address age-dependent measles transmission. Here we described the details about how the four types of contact patterns in this study were constructed.

(i) *POLYMOD matrix*

The original contact data were extracted from the POLYMOD study [1], a large-scale diary-based survey across eight European countries. The physical contact matrix for Great Britain was previously used in the DynaMICE model to approximate the general pattern of age-dependent contact across different settings [10]. In obtaining the matrix ($\boldsymbol{C}_{a,a'}$) ready for use in DynaMICE, we first expanded the original POLYMOD matrix for fine age structure. We then conducted pairwise adjustment [11] to ensure the reciprocity of contact pairs over the simulation period between 2000–2050, based on the time-varying country-specific demographics [9]. Finally, the matrix was standardised by its largest eigenvalue ('POLYMOD' in in Figure S2).

(ii) *Synthetic matrix*

Synthetic contact matrices were built on the POLYMOD surveys and adjusted for household structure, labor participation, school enrolment, and other country-specific characteristics [2]. With the adjustment on country-specific characteristics, synthetic matrices are likely to better represent the contact pattern in countries where empirical contact studies are not available. We followed the same approaches as used in processing the POLYMOD matrix for age structure expansion, reciprocity adjustment, and standardisation ('Synthetic' in Figure S2).

(iii) *Proportional matrix*

We assumed that the contact probability with a particular age group depends on the proportion of population in a specific age group. The proportional mixing matrix is presented as:

$$\boldsymbol{C}_{a,a'}^{(prp)} = \begin{bmatrix} \frac{N_1}{N} & \cdots & \frac{N_{a'}}{N} & \cdots & \frac{N_{254}}{N} \\ \vdots & \ddots & \vdots & \ddots & \vdots \\ \frac{N_1}{N} & \cdots & \frac{N_{a'}}{N} & \cdots & \frac{N_{254}}{N} \end{bmatrix}$$

, where $N_{a'}$ denotes the population size of age a' and N denotes the total population across all ages. For each country, we applied a time-varying matrix according to the age distribution over the simulation years. Based on the proportional mixing, the force of infection matrix is identical across age groups:

$$\lambda_{a,t}^{(prp)} = \omega_t R_0 g \sum_{a'=1}^{a'=254} \boldsymbol{C}_{a,a'}^{(prp)} \frac{(I_{a',t}+VI_{a',t}+V2I_{a',t}+V3I_{a',t})}{N_{a',t}} = \omega_t R_0 g \frac{\sum_{a'=1}^{a'=254}(I_{a',t}+VI_{a',t}+V2I_{a',t}+V3I_{a',t})}{\sum_{a'=1}^{a'=254} N_{a',t}}.$$

This is also known as 'homogeneous mixing' contact pattern.

(iv) *Uniform matrix*

We assumed equal contact probabilities for each yearly age from 0 to 100 years old, and further divided the contact probability by 52 for the weekly age groups. The uniform mixing matrix is written as:

$$\boldsymbol{C}_{a,a'}^{(uni)} = \begin{bmatrix} \underbrace{\begin{matrix} \frac{1}{101\times52} & \cdots & \frac{1}{101\times52} \\ \vdots & \ddots & \vdots \\ \frac{1}{101\times52} & \cdots & \frac{1}{101\times52} \end{matrix}}_{\substack{weekly\ age \\ 0-2\ years\ old}} & \underbrace{\begin{matrix} \frac{1}{101} & \cdots & \frac{1}{101} \\ \vdots & \ddots & \vdots \\ \frac{1}{101} & \cdots & \frac{1}{101} \end{matrix}}_{\substack{yearly\ age \\ 3-100\ years\ old}} \end{bmatrix}.$$

There is no age dependency in mixing and the force of infection is identical across age groups and countries.

Figure S1. Case-fatality risks using Wolfson's and Portnoy's approaches, 2000–2050. In Wolfson's approach, we assumed CFRs for over five years old to be half of that for under five years old [12]. In the Portnoy's approach [13], time-varying CFRs were obtained using a statistical model and assumed to remain constant after 2018. In most countries except Pakistan, the updated CFR estimates using Portnoy's method appear to be relatively lower at a smaller scale.

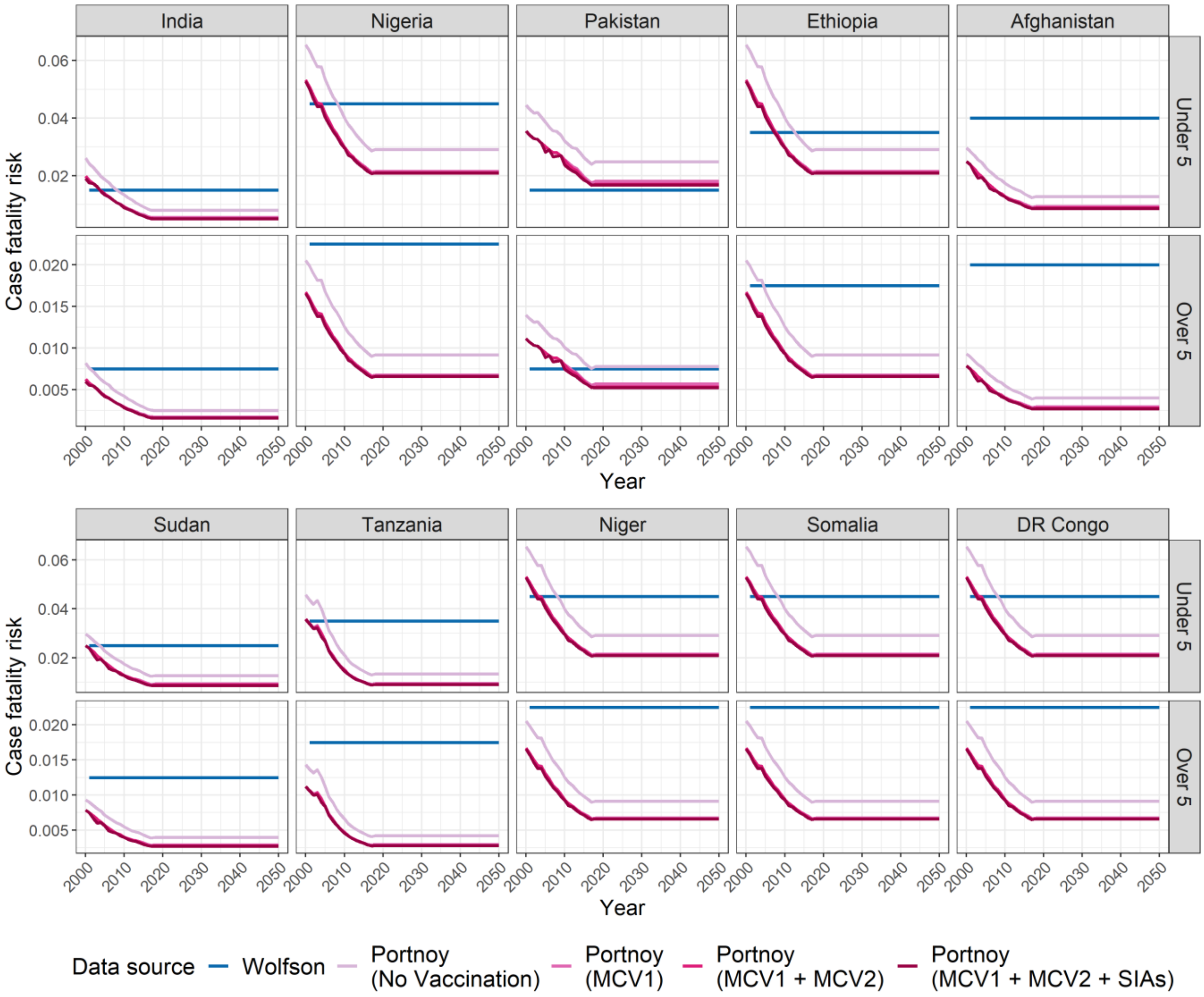

Figure S2. Standardised social contact matrices in ten high-burden countries. POLYMOD Great Britain [1] and country-specific synthetic matrices [2] used in the study are presented. Note that there is no available data for Somalia and thus the synthetic matrix for Ethiopia was adopted considering the geographical proximity and similar socio-behavioural dynamics. For comparison, each matrix is standardised by dividing by its largest eigenvalue.

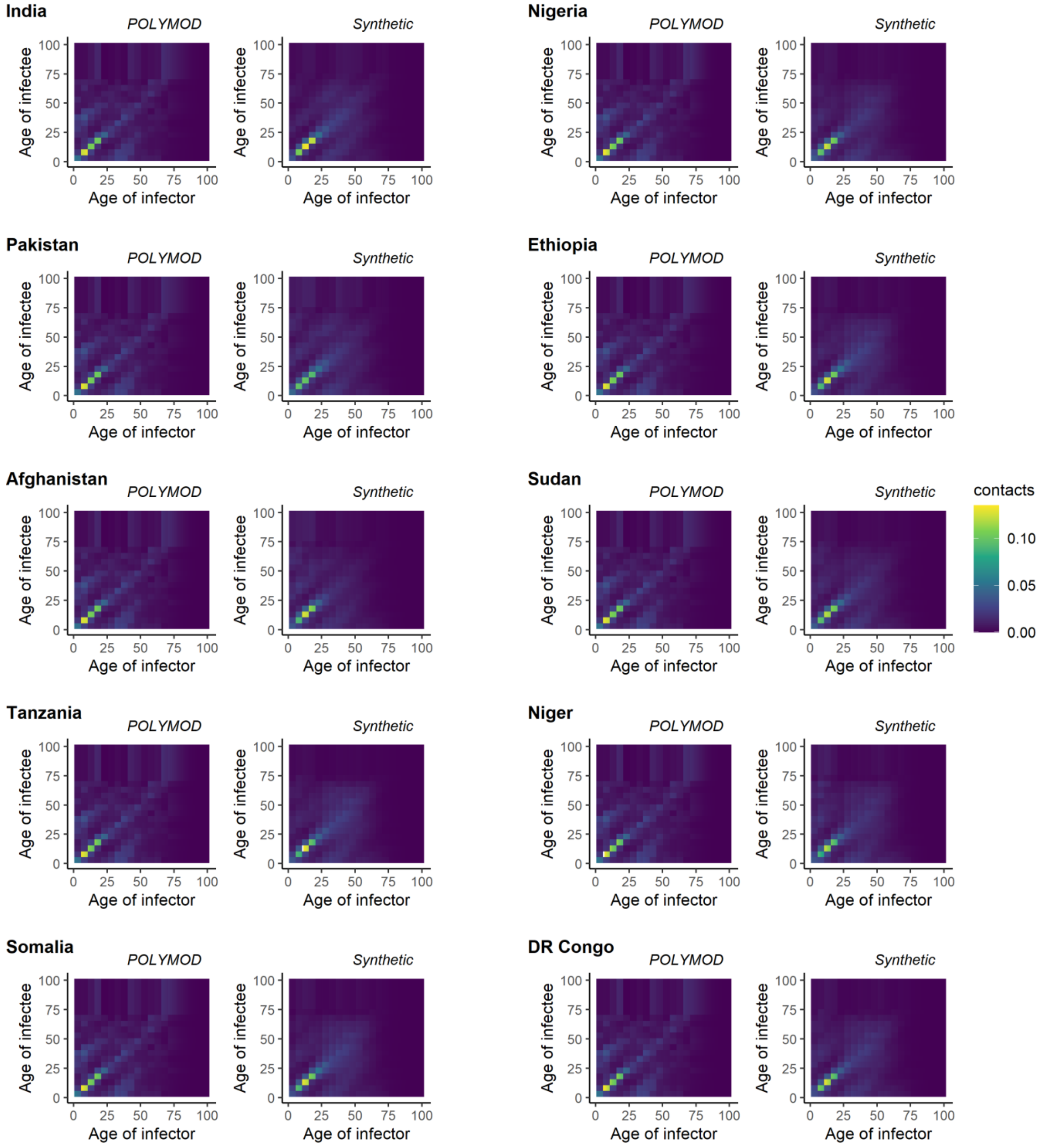

References

1. Mossong J, Hens N, Jit M, Beutels P, Auranen K, Mikolajczyk R, Massari M, Salmaso S, Tomba GS, Wallinga J *et al*: **Social contacts and mixing patterns relevant to the spread of infectious diseases**. *PLoS Med* 2008, **5**(3):e74.
2. Prem K, van Zandvoort K, Klepac P, Eggo RM, Davies NG, Centre for the Mathematical Modelling of Infectious Diseases COVID-19 Working Group, Cook AR, Jit M: **Projecting contact matrices in 177 geographical regions: an update and comparison with empirical data for the COVID-19 era**. *Plos Comput Bio* 2021, **17**(7):e1009098.
3. Guerra FM, Bolotin S, Lim G, Heffernan J, Deeks SL, Li Y, Crowcroft NS: **The basic reproduction number (R0) of measles: a systematic review**. *Lancet Infect Dis* 2017, **17**(12):e420-e428.
4. Strebel PM, Papania MJ, Gastañaduy PA, Goodson JL: **37 - Measles Vaccines**. In: *Plotkin's Vaccines (Seventh Edition).* edn. Edited by Plotkin SA, Orenstein WA, Offit PA, Edwards KM: Elsevier; 2018: 579-618.e521.
5. Sudfeld CR, Navar AM, Halsey NA: **Effectiveness of measles vaccination and vitamin A treatment**. *Int J Epidemiol* 2010, **39 Suppl 1**:i48-55.
6. Hughes SL, Bolotin S, Khan S, Li Y, Johnson C, Friedman L, Tricco AC, Hahne SJM, Heffernan JM, Dabbagh A *et al*: **The effect of time since measles vaccination and age at first dose on measles vaccine effectiveness - A systematic review**. *Vaccine* 2020, **38**(3):460-469.
7. World Health Organization: **Immunization data portal**. In.; 2021.
8. World Health Organization: **Summary of Measles-Rubella Supplementary Immunization Activities, 2000-2020**. In. Edited by Immunization VaBD; 2020.
9. United Nations Deparatment of Economic and Social Affairs: **World Population Prospects**. In.; 2019.
10. Verguet S, Johri M, Morris SK, Gauvreau CL, Jha P, Jit M: **Controlling measles using supplemental immunization activities: a mathematical model to inform optimal policy**. *Vaccine* 2015, **33**(10):1291-1296.
11. Arregui S, Aleta A, Sanz J, Moreno Y: **Projecting social contact matrices to different demographic structures**. *Plos Comput Biol* 2018, **14**(12):e1006638.
12. Wolfson LJ, Grais RF, Luquero FJ, Birmingham ME, Strebel PM: **Estimates of measles case fatality ratios: a comprehensive review of community-based studies**. *Int J Epidemiol* 2009, **38**(1):192-205.
13. Portnoy A, Jit M, Ferrari M, Hanson M, Brenzel L, Verguet S: **Estimates of case-fatality ratios of measles in low-income and middle-income countries: a systematic review and modelling analysis**. *Lancet Glob Health* 2019, **7**(4):e472-e481.
