# Supplementary material for: Effect of evidence updates on key determinants of measles vaccination impact: a DynaMICE modelling study in ten high-burden countries
Source: BMC Med. 2021 Nov 17;19:281. doi: 10.1186/s12916-021-02157-4 (PMC8594955; doi:10.1186/s12916-021-02157-4)
Supplement: Supplementary file 2 — Additional file 2: Supplementary results. Table S2–Calendar year at which low measles incidence is achieved. Table S3–National and total measles burden by selected scenarios and vaccine strategies. Table S4–Averted measles burden in consideration of COVID-19 related disruptions. Figure S3–Model estimates under different assumptions for SIA delivery to zero-dose children. Figure S4–Model estimates of measles cases in the ‘base’ and ‘full-update’ scenarios. Figure S5–Model estimates of (A) measles cases and (B) deaths over 2000–2050 under different assumptions of R0. Figure S6–Model estimates of (A) measles cases and (B) deaths over 2000–2050 under different assumptions of R0, assuming no SIAs after 2019. [file 12916_2021_2157_MOESM2_ESM.pdf]

Additional file 2

Supplementary results for the manuscript entitled "Effect of evidence updates on key determinants of measles vaccination impact: a DynaMICE modelling study in ten high-burden countries"

Han Fu[1], Kaja Abbas[1,2,3], Petra Klepac[1,4], Kevin van Zandvoort[1], Hira Tanvir[1], Allison Portnoy[5], Mark Jit[1,6,7]

[1]Department of Infectious Disease Epidemiology, London School of Hygiene & Tropical Medicine, London, UK

[2]Public Health Foundation of India, New Delhi, India

[3]International Vaccine Institute, Seoul, South Korea

[4]Department of Applied Mathematics and Theoretical Physics, University of Cambridge, Cambridge, UK

[5]Center for Health Decision Science, Harvard T.H. Chan School of Public Health, Boston, MA, USA

[6]Modelling and Economics Unit, Public Health England, London, UK

[7]School of Public Health, University of Hong Kong, Hong Kong, SAR, China

Contents

Table S2. Calendar year at which low measles incidence is achieved. 2

Table S3. National and total measles burden by selected scenarios and vaccine strategies. 4

Table S4. Averted measles burden in consideration of COVID-19 related disruptions. 8

Figure S3. Model estimates under different assumptions for SIA delivery to zero-dose children. 9

Figure S4. Model estimates of measles cases in the 'base' and 'full-update' scenarios. 10

Figure S5. Model estimates of (A) measles cases and (B) deaths over 2000–2050 under different assumptions of R_0. 11

Figure S6. Model estimates of (A) measles cases and (B) deaths over 2000–2050 under different assumptions of R_0, assuming no SIAs after 2019. 12

References 12

Table S2. Calendar year at which low measles incidence is achieved. Low incidence is defined as an annual incidence rate of < 1 case per million population for at least three consecutive years, denoting conditions that may be consistent with ability to achieve elimination. Model results based on the 'base' and 'full-update' scenarios (denoted as B and F, respectively) and with a vaccination strategy including MCV1, MCV2, and SIAs are presented for the 10 high measles burden countries – India, Nigeria, Pakistan, Ethiopia, Afghanistan, Sudan, Tanzania, Niger, Somalia, and DR Congo.

Country and scenario		R_0 = 12	R_0 = 16 ('base' scenario)	R_0 = 20	R_0 = 24
India	B	2022	2022	Not achievable before 2050 (once verified in 2023, but reintroduced in 2025)	Not achievable before 2050
	F	2021	2021	Not achievable before 2050 (once verified in 2021, but reintroduced in 2032)	Not achievable before 2050 (once verified in 2022, but reintroduced in 2026)
Nigeria	B	2010	2041 (once verified in 2015, but reintroduced in 2025)	2044 (once verified in 2021, but reintroduced in 2022)	Not achievable before 2050
	F	2010	2047 (once verified in 2010, but reintroduced in 2028)	Not achievable before 2050 (once verified in 2020, but reintroduced in 2022)	Not achievable before 2050 (once verified in 2021, but reintroduced in 2022)
Pakistan	B	2011	2012	2017	2018
	F	2011	2012	2018	2018
Ethiopia	B	2011	2027 (once verified in 2011, but reintroduced in 2019)	2036	2042
	F	2012	2011	2027 (once verified in 2010, but reintroduced in 2019)	2024
Afghanistan	B	2016 (once verified in 2010, but reintroduced in 2011)	2018	2037	2045
	F	2006	2016	2030 (once verified in 2016, but reintroduced in 2023)	2040
Sudan	B	2008	2009	2009	2012
	F	2008	2008	2009	2010
Tanzania	B	2005	2005	2011	2050 (once verified in 2009, but reintroduced in 2028)

	F	2005	2005	2048<br>(once verified in 2012, but reintroduced in 2032)	Not achievable before 2050<br>(once verified in 2009, but reintroduced in 2040)
Niger	B	2012	2012	2012	2018<br>(once verified in 2013, but reintroduced in 2014)
	F	2008	2012	2012	2012
Somalia	B	Not achievable before 2050<br>(once verified in 2011, but reintroduced in 2022)	Not achievable before 2050<br>(once verified in 2014, but reintroduced in 2021)	Not achievable before 2050<br>(once verified in 2015, but reintroduced in 2020)	Not achievable before 2050
	F	Not achievable before 2050<br>(once verified in 2011, but reintroduced in 2024)	Not achievable before 2050<br>(once verified in 2011, but reintroduced in 2022)	Not achievable before 2050<br>(once verified in 2013, but reintroduced in 2021)	Not achievable before 2050<br>(once verified in 2014, but reintroduced in 2021)
DR Congo	B	Not achievable before 2050<br>(once verified in 2010, but reintroduced in 2021)	Not achievable before 2050<br>(once verified in 2016, but reintroduced in 2020)	Not achievable before 2050<br>(once verified in 2016, but reintroduced in 2018)	Not achievable before 2050<br>(once verified in 2016, but reintroduced in 2018)
	F	Not achievable before 2050<br>(once verified in 2016, but reintroduced in 2022)	Not achievable before 2050<br>(once verified in 2016, but reintroduced in 2021)	Not achievable before 2050<br>(once verified in 2016, but reintroduced in 2020)	Not achievable before 2050<br>(once verified in 2016, but reintroduced in 2018)

Table S3. National and total measles burden by selected scenarios and vaccine strategies. Cumulative measles cases, deaths, and DALYs over 2000–2050 are presented in millions in India, Nigeria, Pakistan, Ethiopia, Afghanistan, Sudan, Tanzania, Niger, Somalia, DR Congo, and in total. Details of data sources for key determinants assumed for each scenario can be found in Table 1 in the main text.

Scenarios/ country	No vaccination			MCV1			MCV1 + MCV2			MCV1 + MCV2 + SIAs		
	cases	deaths	DALYs	cases	deaths	DALYs	cases	deaths	DALYs	cases	deaths	DALYs
Base												
India	1183	17	1222	344	4.3	293	205	2.7	180	168	2.3	150
Nigeria	373	16	959	109	4.2	237	98	3.8	211	15	0.64	32
Pakistan	274	4.0	279	62	0.76	51	34	0.43	29	13	0.17	11
Ethiopia	173	5.9	406	63	1.9	128	54	1.7	108	7.4	0.24	14
Afghanistan	57	2.2	149	24	0.85	56	21	0.74	48	2.5	0.097	5.8
Sudan	70	1.7	115	12	0.23	15	4.3	0.092	5.7	1.5	0.035	2.1
Tanzania	112	3.8	259	16	0.42	27	3.9	0.10	6.0	0.093	0.0025	0.13
Niger	59	2.6	171	10	0.38	24	4.5	0.18	10	2.1	0.092	5.2
Somalia	33	1.5	89	14	0.58	35	14	0.57	34	9.1	0.37	22
DR Congo	175	7.7	493	51	1.9	121	47	1.8	114	33	1.3	79
Total	2509	63	4142	706	16	987	486	12	745	252	5.2	322
(A) CFR, Portnoy's method												
India	-	12	867	-	2.6	179	-	2.1	140	-	1.9	126
Nigeria	-	12	685	-	2.5	137	-	2.3	125	-	0.65	33
Pakistan	-	7.3	506	-	1.1	73	-	0.73	49	-	0.35	23
Ethiopia	-	5.6	380	-	1.5	96	-	1.3	83	-	0.29	17
Afghanistan	-	0.81	54	-	0.25	16	-	0.22	14	-	0.049	3.0
Sudan	-	0.98	66	-	0.11	7.0	-	0.064	4.0	-	0.030	1.9
Tanzania	-	1.8	116	-	0.13	8.2	-	0.054	3.1	-	0.0023	0.12
Niger	-	1.8	119	-	0.23	14	-	0.14	8.3	-	0.087	4.8
Somalia	-	1.0	63	-	0.33	19	-	0.31	19	-	0.20	12
DR Congo	-	5.5	349	-	1.1	67	-	1.0	62	-	0.66	41
Total	-	49	3205	-	9.9	616	-	8.3	507	-	4.2	261
(B) Contact pattern, synthetic matrices												
India	1186	17	1168	328	3.7	248	204	2.4	160	160	1.9	129
Nigeria	369	16	927	106	3.9	217	95	3.5	194	14	0.57	29
Pakistan	274	4.0	276	61	0.72	49	33	0.42	28	12	0.16	11
Ethiopia	171	5.6	382	60	1.6	108	51	1.4	92	4.0	0.12	6.9
Afghanistan	56	2.1	143	23	0.77	50	20	0.67	44	1.9	0.065	4.0
Sudan	69	1.7	112	11	0.21	13	4.3	0.086	5.4	1.4	0.031	1.9
Tanzania	110	3.6	246	14	0.34	22	3.8	0.093	5.4	0.11	0.0028	0.15
Niger	58	2.4	162	9.7	0.33	21	4.4	0.17	9.7	1.2	0.048	2.7
Somalia	32	1.3	83	13	0.49	30	13	0.48	29	7.5	0.27	16

DR Congo	171	7.1	461	48	1.7	104	45	1.6	98	30	1.0	64
Total	2497	60	3960	674	14	861	473	11	664	232	4.2	264
(B’) Contact pattern, proportional mixing												
India	1133	14	1004	277	3.0	195	178	2.0	132	122	1.5	96
Nigeria	362	15	894	100	3.7	207	90	3.4	187	14	0.59	30
Pakistan	263	3.5	245	51	0.57	38	28	0.34	23	11	0.14	9.0
Ethiopia	166	5.3	365	55	1.6	103	47	1.4	88	6.7	0.22	13
Afghanistan	54	2.0	133	21	0.70	45	18	0.61	39	2.1	0.076	4.6
Sudan	67	1.6	105	10	0.19	12	3.8	0.079	4.9	1.4	0.031	1.9
Tanzania	109	3.6	241	14	0.35	23	3.6	0.098	5.6	0.085	0.0023	0.12
Niger	57	2.5	164	10	0.37	23	4.8	0.19	11	2.1	0.091	5.1
Somalia	32	1.4	84	13	0.53	32	13	0.51	30	7.8	0.30	18
DR Congo	169	7.2	462	46	1.7	107	43	1.6	100	29	1.1	66
Total	2411	57	3697	597	13	784	429	10	621	196	4.0	243
(B’’) Contact pattern, uniform mixing												
India	1189	13	916	311	2.9	178	211	2.0	123	123	1.2	78
Nigeria	332	12	672	80	2.4	129	73	2.2	118	11	0.35	17
Pakistan	261	3.0	203	50	0.47	29	30	0.29	18	9.4	0.095	6.0
Ethiopia	161	4.3	290	49	1.2	72	42	1.0	62	0.41	0.0099	0.54
Afghanistan	53	1.6	107	19	0.52	32	17	0.45	28	0.97	0.029	1.7
Sudan	64	1.2	82	8.8	0.14	8.3	4.4	0.071	4.1	1.1	0.020	1.2
Tanzania	100	2.7	180	10	0.23	14	4.0	0.093	4.8	0.17	0.0038	0.19
Niger	50	1.8	116	7.0	0.21	12	4.2	0.13	7.4	0.90	0.031	1.7
Somalia	29	1.0	62	11	0.33	19	10	0.32	19	4.5	0.14	8.1
DR Congo	155	5.4	345	36	1.1	65	34	1.1	62	18	0.54	32
Total	2395	46	2973	582	9.5	560	430	7.6	445	170	2.5	147
(C) First-dose vaccine efficacy, linear trend by age												
India	-	-	-	432	5.6	384	236	3.1	209	187	2.6	171
Nigeria	-	-	-	136	5.4	305	120	4.7	265	22	0.89	46
Pakistan	-	-	-	85	1.1	73	39	0.51	34	15	0.20	13
Ethiopia	-	-	-	74	2.3	155	60	1.9	123	12	0.38	23
Afghanistan	-	-	-	27	0.98	65	23	0.82	53	2.9	0.11	6.9
Sudan	-	-	-	18	0.37	24	5.6	0.12	7.6	1.7	0.040	2.4
Tanzania	-	-	-	26	0.73	49	6.4	0.18	11	0.24	0.0062	0.34
Niger	-	-	-	15	0.58	37	6.2	0.24	14	2.3	0.10	5.6
Somalia	-	-	-	16	0.67	40	16	0.64	38	11	0.43	26
DR Congo	-	-	-	63	2.5	157	59	2.3	145	42	1.6	104
Total	-	-	-	893	20	1288	570	15	900	295	6.4	399
(D) SIA delivery to zero-dose population, dependency on previous vaccination												

India	-	-	-	-	-	-	-	-	-	160	2.2	144
Nigeria	-	-	-	-	-	-	-	-	-	15	0.64	32
Pakistan	-	-	-	-	-	-	-	-	-	13	0.17	11
Ethiopia	-	-	-	-	-	-	-	-	-	7.1	0.23	14
Afghanistan	-	-	-	-	-	-	-	-	-	2.7	0.10	5.9
Sudan	-	-	-	-	-	-	-	-	-	1.5	0.035	2.1
Tanzania	-	-	-	-	-	-	-	-	-	0.094	0.0025	0.14
Niger	-	-	-	-	-	-	-	-	-	2.1	0.091	5.1
Somalia	-	-	-	-	-	-	-	-	-	9.0	0.36	22
DR Congo	-	-	-	-	-	-	-	-	-	32	1.2	77
Total	-	-	-	-	-	-	-	-	-	243	5.0	313
(D’) SIA delivery to zero-dose population, dependency on previous vaccination, excluding the largest survey												
India	-	-	-	-	-	-	-	-	-	163	2.2	146
Nigeria	-	-	-	-	-	-	-	-	-	15	0.63	32
Pakistan	-	-	-	-	-	-	-	-	-	13	0.17	11
Ethiopia	-	-	-	-	-	-	-	-	-	7.2	0.24	14
Afghanistan	-	-	-	-	-	-	-	-	-	2.5	0.095	5.7
Sudan	-	-	-	-	-	-	-	-	-	1.6	0.035	2.1
Tanzania	-	-	-	-	-	-	-	-	-	0.090	0.0024	0.13
Niger	-	-	-	-	-	-	-	-	-	2.1	0.091	5.1
Somalia	-	-	-	-	-	-	-	-	-	9.0	0.36	22
DR Congo	-	-	-	-	-	-	-	-	-	33	1.2	78
Total	-	-	-	-	-	-	-	-	-	246	5.1	316
(D’’) SIA delivery to zero-dose population, 7.7% never reached												
India	-	-	-	-	-	-	-	-	-	192	2.5	170
Nigeria	-	-	-	-	-	-	-	-	-	56	2.2	120
Pakistan	-	-	-	-	-	-	-	-	-	28	0.36	24
Ethiopia	-	-	-	-	-	-	-	-	-	46	1.4	91
Afghanistan	-	-	-	-	-	-	-	-	-	6.4	0.24	15
Sudan	-	-	-	-	-	-	-	-	-	2.9	0.063	3.9
Tanzania	-	-	-	-	-	-	-	-	-	0.87	0.023	1.3
Niger	-	-	-	-	-	-	-	-	-	2.4	0.10	5.8
Somalia	-	-	-	-	-	-	-	-	-	13	0.52	31
DR Congo	-	-	-	-	-	-	-	-	-	44	1.7	104
Total	-	-	-	-	-	-	-	-	-	390	9.1	566
Full-update												
India	1186	12	814	412	2.7	180	228	2.0	133	168	1.7	111
Nigeria	369	11	656	131	2.8	154	116	2.5	136	15	0.62	31
Pakistan	274	7.2	500	83	1.4	96	38	0.82	55	14	0.39	25

Ethiopia	171	5.2	350	70	1.4	92	57	1.2	76	4.6	0.16	9.6
Afghanistan	56	0.77	51	26	0.25	16	22	0.22	14	2.3	0.037	2.2
Sudan	69	0.95	64	17	0.14	9.4	5.7	0.074	4.6	1.6	0.030	1.8
Tanzania	110	1.7	109	24	0.18	12	6.3	0.075	4.4	0.10	0.0023	0.12
Niger	58	1.7	111	14	0.28	17	5.9	0.16	9.0	1.3	0.053	2.9
Somalia	32	0.95	58	15	0.30	18	14	0.29	17	8.7	0.16	9.8
DR Congo	171	5.0	319	59	1.1	69	55	1.0	64	37	0.62	39
Total	2497	46	3031	851	11	664	548	8.3	513	253	3.8	233

Table S4. Averted measles burden in consideration of COVID-19 related disruptions. As presented in Table 2 in the main text, total vaccine-averted burden cases, deaths, DALYs and proportionate change from the 'base' scenario (parentheses) in ten high measles burden countries over 2000–2050 are shown. These model estimates were generated under the modified projections for coverage and case-fatality risk estimates affected by the COVID-19 pandemic. All model scenarios were conducted under the assumption of $R_0 = 16$.

**Scenarios**	**MCV1**			**MCV1 + MCV2**			**MCV1 + MCV2 + SIAs**		
	**Cases, millions**	**Deaths, millions**	**DALYs, millions**	**Cases, millions**	**Deaths, millions**	**DALYs, millions**	**Cases, millions**	**Deaths, millions**	**DALYs, millions**
Base	1790 (ref)	46.9 (ref)	3127 (ref)	2006 (ref)	50.3 (ref)	3362 (ref)	2248 (ref)	57.4 (ref)	3798 (ref)
(A) CFR, Portnoy's method	-	39.1 (-16.7%)	2576 (-17.6%)	-	40.6 (-19.3%)	2683 (-20.2%)	-	44.8 (-22.1%)	2935 (-22.7%)
(B) Contact pattern, synthetic matrices	1813 (1.2%)	46.1 (-1.7%)	3075 (-1.7%)	2009 (0.15%)	48.9 (-2.8%)	3268 (-2.8%)	2258 (0.47%)	55.7 (-3.0%)	3683 (-3.0%)
(B') Contact pattern, proportional mixing	1804 (0.76%)	43.5 (-7.2%)	2891 (-7.5%)	1969 (-1.9%)	45.9 (-8.7%)	3050 (-9.3%)	2210 (-1.7%)	52.4 (-8.7%)	3443 (-9.4%)
(B'') Contact pattern, uniform mixing	1805 (0.80%)	36.2 (-22.7%)	2399 (-23.3%)	1952 (-2.7%)	38.1 (-24.4%)	2510 (-25.3%)	2211 (-1.6%)	43.4 (-24.5%)	2818 (-25.8%)
(C) Age-dependent vaccine efficacy, linear trend	1605 (-10.3%)	42.4 (-9.6%)	2828 (-9.6%)	1922 (-4.2%)	47.8 (-4.9%)	3206 (-4.7%)	2201 (-2.1%)	56.2 (-2.2%)	3717 (-2.1%)
(D) SIA delivery to zero-dose population, dependency on previous vaccination	-	-	-	-	-	-	2257 (0.42%)	57.6 (0.29%)	3809 (0.29%)
(D') SIA delivery to zero-dose population, dependency on previous vaccination, excluding the largest survey	-	-	-	-	-	-	2257 (0.42%)	57.7 (0.43%)	3813 (0.39%)
(D'') SIA delivery to zero-dose population, 7.7% never reached	-	-	-	-	-	-	2104 (-6.4%)	53.3 (-7.2%)	3545 (-6.7%)
Full-update	1636 (-8.6%)	35.7 (-23.8%)	2357 (-24.6%)	1933 (-3.7%)	37.9 (-24.8%)	2505 (-25.5%)	2237 (0.50%)	42.5 (-26.0%)	2791 (-26.5%)

Figure S3. Model estimates under different assumptions for SIA delivery to zero-dose children. Measles burden estimates were conducted based on the 'base' scenario, under the assumption of R_0 = 16 and with the implementation of MCV1, MCV2, and SIAs. Four different approaches for distributing SIA doses between zero-dose children and already-vaccinated children. Apart from the other three approaches, assuming that 7.7% of children are never reached for any MCV dose [1] and randomly delivering SIA doses to the rest would result in greater and more persistent measles burden over 2000–2050.

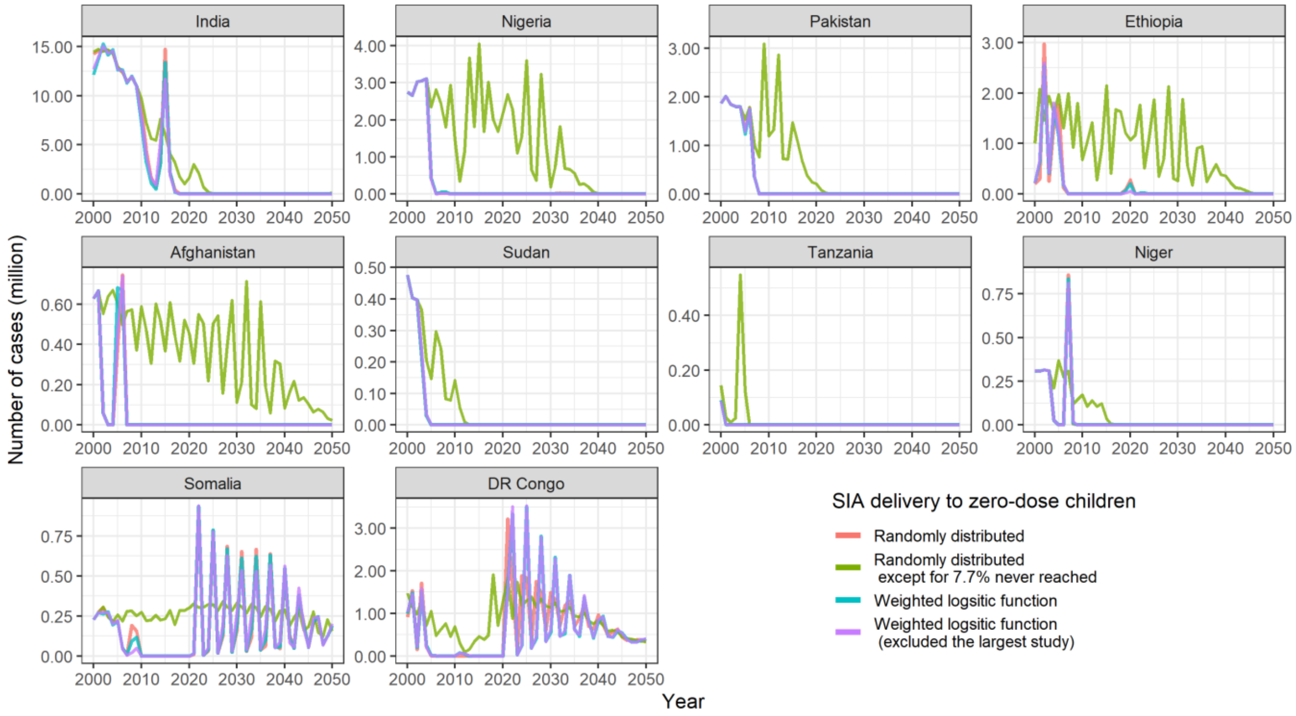

Figure S4. Model estimates of measles cases in the 'base' and 'full-update' scenarios. Number of measles cases were estimated under the assumption of R_0 = 12, 16, 20, and 24, and with the implementation of MCV1, MCV2, and SIAs. Model-based estimates were used to compare with data from Institute for Health Metrics and Evaluation [2] and notifications from World Health Organization [3].

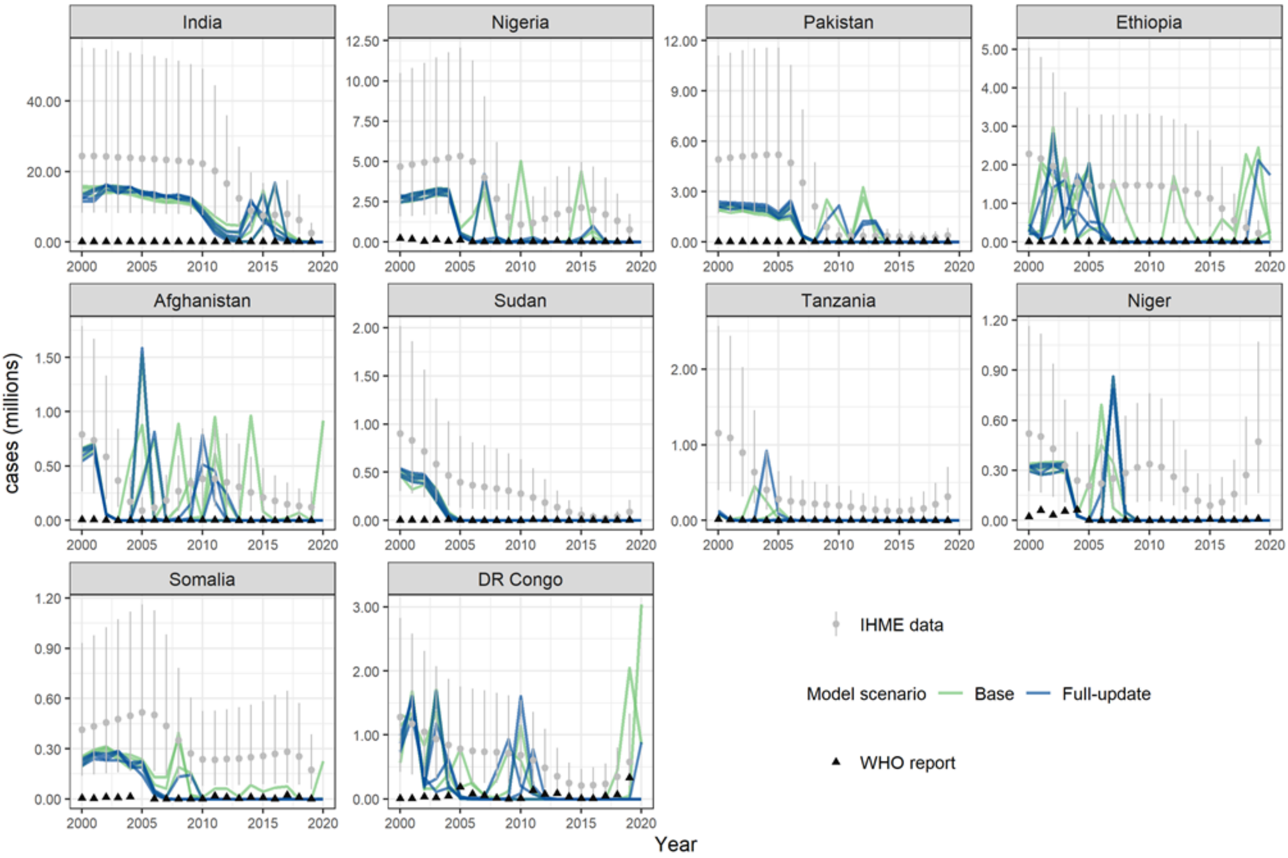

Figure S5. Model estimates of (A) measles cases and (B) deaths over 2000–2050 under different assumptions of R_0. Measles burden estimates based on the 'full-update' scenario and the vaccination strategy that includes MCV1, MCV2, and SIAs are used for comparing with estimates from IHME [2] and notifications from WHO [3].

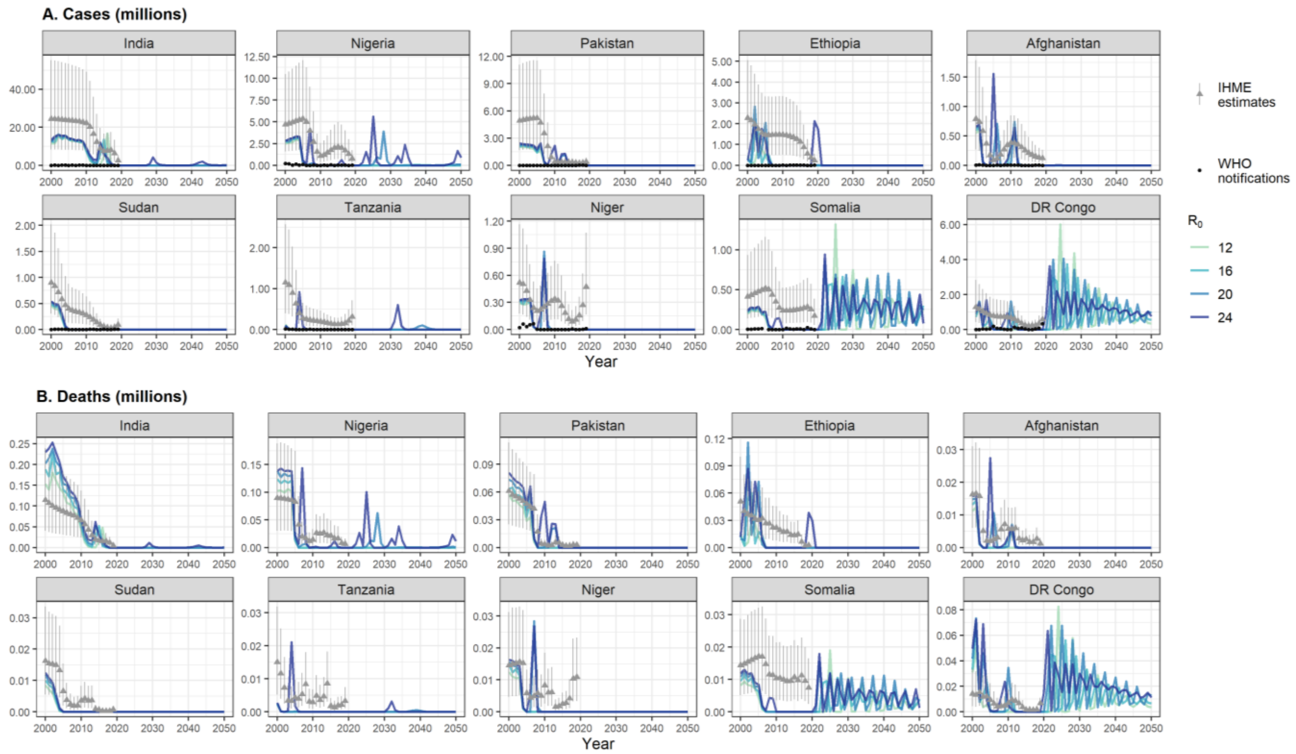

Figure S6. Model estimates of (A) measles cases and (B) deaths over 2000–2050 under different assumptions of R_0, assuming no SIAs after 2019. As described in the caption of Supplementary Figure 6, but no SIAs are implemented after 2020 (0% coverage) in this projection.

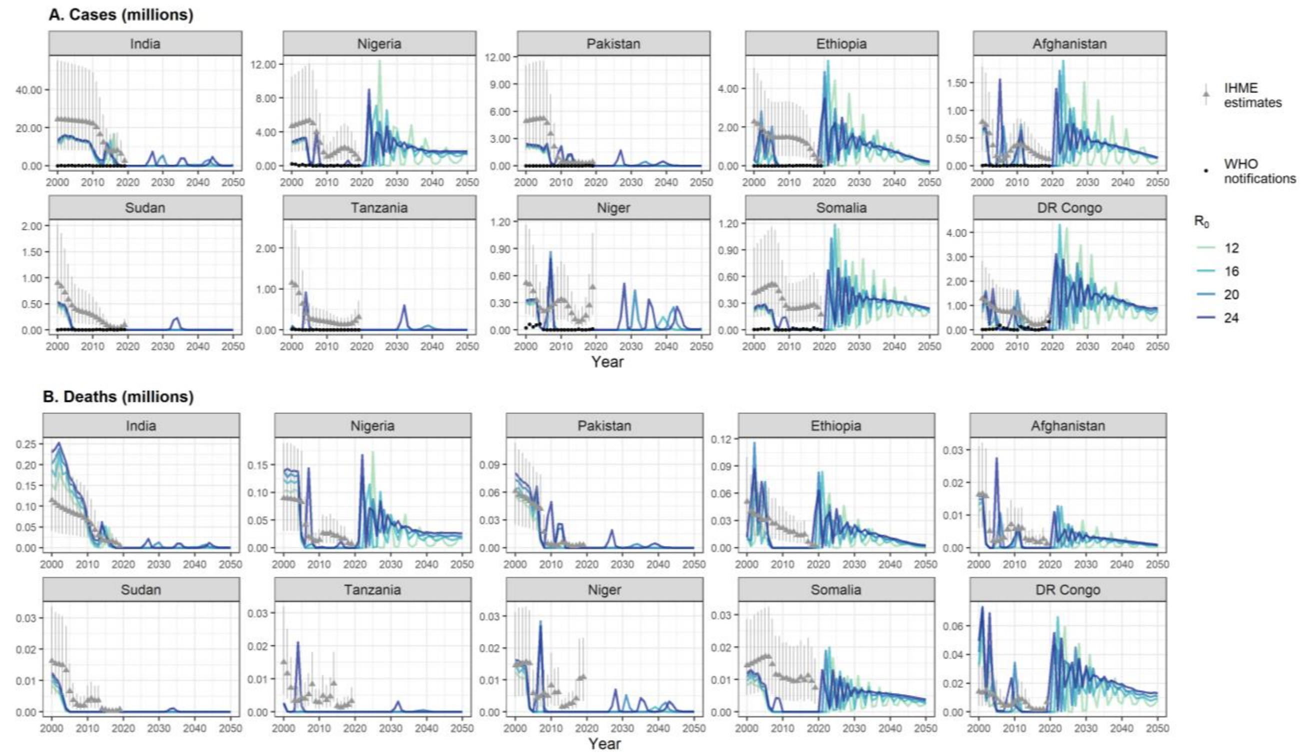

References

1. Cata-Preta BO, Santos TM, Mengistu T, Hogan DR, Barros AJD, Victora CG: **Zero-dose children and the immunisation cascade: Understanding immunisation pathways in low and middle-income countries**. *Vaccine* 2021, **39**(32):4564-4570.
2. Global Burden of Disease Collaborative Network: **Global Burden of Disease Study 2019 (GBD 2019) Results**. In. Seattle, United States: Institute for Health Metrics and Evaluation (IHME); 2020.
3. World Health Organization: **Provisional monthly measles and rubella data**. In: *2011-2020.* 2021.
